# Dimethyl Sulfoxide Conditions Induced Pluripotent Stem Cells for more Efficient Nephron Progenitor and Kidney Organoid Differentiation

**DOI:** 10.1007/s12015-025-10971-z

**Published:** 2025-09-18

**Authors:** Helen Kearney, Aleksandra Rak-Raszewska, Adrián Seijas-Gamardo, Enrique Escarda-Castro, Florian Caiment, Paul Wieringa, Lorenzo Moroni, Carlos Mota

**Affiliations:** 1 MERLN Institute for Technology-Inspired Regenerative Medicine, Maastricht University, Maastricht, 6229 ER the Netherlands; 2 Translational Genomics, GROW Research Institute for Oncology and Reproduction, Maastricht University, Maastricht, 6229 ER the Netherlands

**Keywords:** Kidney organoids, Induced-pluripotent stem cells, Dimethyl sulfoxide, Differentiation potential, Nephron progenitor cells, Differentiation protocol efficiency

## Abstract

The field of human induced pluripotent stem cells (hiPSCs) has seen significant progress since the discovery of reprogramming somatic cells using the transcription factors Oct4, Sox2, Klf4, and c-Myc. hiPSCs are similar to embryonic stem cells in a primed state of pluripotency and have the potential to differentiate into any adult human cell type, offering a versatile tool for research and potential therapeutic applications. However, the efficiency of differentiation protocols for generating complex structures with multiple cell types, Like kidney organoids, remains a challenge. This study investigates the impact of treating hiPSCs with a low-dose dimethyl sulfoxide to enhance kidney organoid differentiation using the stepwise 2D monolayer-based protocol developed by Morizane et al. 2017. We found that treating hiPSCs with 1–2% DMSO affects gene expression of pluripotent transcription factors, the epigenetic landscape, and hiPSC colony morphology. Our findings also suggest DMSO treatment enhances the expression of the key metanephric mesenchyme nephron progenitor marker, SIX2 after 9 days of kidney organoid differentiation and helps improve hiPSC differentiation protocol efficiency toward the development of tubular kidney organoids. Further research is needed to fully elucidate the mechanisms underlying these effects and refine the differentiation process for potential in vitro research applications in biomedical research and drug development.

**Graphical Abstract:**

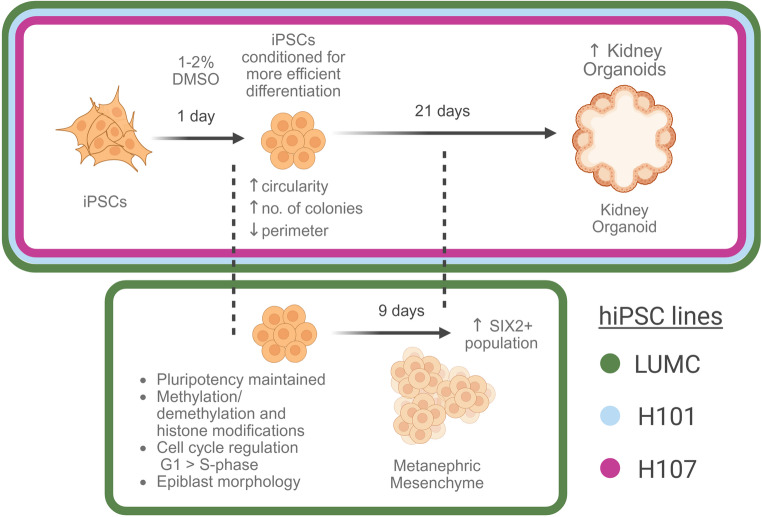

**Supplementary Information:**

The online version contains supplementary material available at 10.1007/s12015-025-10971-z.

## Introduction

In less than two decades since Yamanaka and colleagues first published a protocol to reprogram somatic cells back to a pluripotent state using key transcription factors such as Oct4, Sox2, Klf4, and c-Myc, the field of human induced pluripotent stem cells (hiPSCs) has undergone remarkable advancements [1, 2]. This Nobel-prize winning work has equipped researchers with the tools to induce pluripotency from any individual donor, directing differentiation towards any cell type in the body. Notably, hiPSCs also circumvents many ethical concerns associated with the use of embryonic stem cells, thereby opening new avenues in stem cell biology and its diverse applications [3]. hiPSCs are often considered to be like embryonic stem cells in a “primed state,” representing a more developmentally advanced pluripotent state resembling post-implantation epiblast cells [4, 5]. These cells can self-renew and differentiate into all three germ layers in vitro, but their ability to contribute to chimeras is limited. They grow as flat colonies and require FGF and Activin signalling for maintenance; these signalling pathways support self-renewal and help sustain the pluripotent state of hiPSCs [6, 7]. These factors contribute to why hiPSCs are deemed to be in a more “primed state”.

Recent research has highlighted key growth factors necessary to differentiate hiPSCs into kidney organoids, multicellular structures that closely mimic the fetal kidney development up to the second trimester [8–12]. Kidney organoids contain early-stage nephron structures, including glomeruli, proximal tubule, distal tubule, and collecting ducts, exhibiting a degree of functionality comparable to in vivo tissue. Kidney organoids hold significant promise for nephrotoxicity screening, potentially replacing animal models known for their poor predictive power [13]. However, current protocols for generating kidney organoids still face challenges such as limited efficiency, maturation, and the emergence of off-target cell populations with prolonged culture [14, 15].

Differentiation protocols require transitioning hiPSCs through early developmental stages, from primitive streak to intermediate mesoderm and nephron progenitors before forming kidney organoids [16]. Optimizing each stage of differentiation, particularly the starting hiPSC population, is crucial.

Previous studies show that treating human pluripotent stem cells (hPSCs) with a low concentration of dimethyl sulfoxide (DMSO) prior to directed differentiation increases differentiation across all germ layers. Chetty et al. show that DMSO prevents the phosphorylation of retinoblastoma, halting cells in the G1 phase of the cell cycle via alterations in PI3K pathway signalling, which they found regulates the early transitory states of hPSCs toward differentiation [17, 18]. Additionally, they discovered that genes involved in cytoskeletal dynamics, cilium assembly, and cell adhesion were particularly influenced by DMSO treatment. The integration of these signalling pathways regulates various developmental processes, including proliferation, differentiation, fate determination, apoptosis, migration, adhesion, and cell shape, ultimately impacting organogenesis. Other studies demonstrated that the use of low concentrations of DMSO improved differentiation into various cell types [19, 20]. However, limited knowledge is available on the effect of DMSO treatment on the differentiation efficiency of hiPSC-derived kidney organoids.

The primary objective of this study was to examine the effect of treating hiPSCs with a low dose of DMSO for 24 h, and how it can enhance the efficiency of kidney organoid differentiation following a well-established protocol from Morizane et al. [21]. Firstly, we explored the potential impact DMSO has on the “primed state” of our hiPSCs, by measuring markers of pluripotency and examining hiPSC growth and colony morphology changes in real-time over 24 h. Additionally, the downstream effects on kidney organoid differentiation was measured by quantifying the expression of proteins such as SIX2, a marker for metanephric mesenchyme (MM) nephron progenitor cells (NPC) [22], and other characteristic kidney organoid proteins markers, like podocalyxin (PODXL), megalin (LRP2), and GATA-binding protein 3 (GATA3) on the final day of differentiation [8–12].

## Materials and Methods

### Human Induced Pluripotent Stem Cell Culture Maintenance

hiPSC lines LUMC0031iCTRL08; LUMCi004-C (RRID: CVCL_ZA01), referred to hereafter as LUMC, HUMIMC101; TISSUi001-A (RRID: CVCL_WU56), referred to hereafter as H101, and HUMIMC107; TISSUi007-A (RRID: CVCL_WU62), referred to hereafter as H107, were maintained as colonies on 1% Geltrex (Gibco) coated Nunc™ cell culture treated plates (ThermoFisher) with mTeSRplus medium (STEMCELL Technologies) (Table 1). Cells were passaged once the colonies grew to the point where edges were rounded. Cell colonies were dissociated from culture wells using gentle cell dissociation reagent (STEMCELL Technologies) and washed with phosphate buffered saline (PBS) (Gibco) before scraping from surface using a sterile cell Lifter. Colonies were gently broken up by pipetting and reseeded at the desired splitting ratio, normally 1:10.

**Table 1:** HiPSC lines used in this study, with donor information and source cell types

hiPSC line name	Alternative name	Sex of donor	Source cell type	Referred to as
LUMCi004-C (RRID: CVCL_ZA01)	LUMC0031iCTRL08	Female	Urine material	LUMC
TISSUi001-A (RRID: CVCL_WU56)	HUMIMC101	Male	Peripheral Blood Mononuclear Cell	H101
TISSUi007-A (RRID: CVCL_WU62)	HUMIMC107	Male	Peripheral Blood Mononuclear Cell	H107

### Seeding hiPSCs for Differentiation

Cells were differentiated following a previously published protocol for differentiating hiPSC toward MM NPC and kidney organoid formation, and optimized for the LUMC hiPSCs following guidelines in Morizane et al. [21]. Briefly, 1mL Accutase (STEMCELL Technologies) was added to a well of hiPSC and incubated at 37 °C for 10 min. A single cell suspension of hiPSCs resuspended in mTeSRplus medium supplemented with 10 µM Y-27,632 dihydrochloride (Tocris) and seeded onto cell culture treated plates previously coated with 1% geltrex (ThermoFisher) solution according to manufacturer’s guidelines at a specific cell density for each hiPSC Line used: 1× 10^4^ cell/cm^2^ for LUMC, 9 × 10^3^ cell/cm^2^ for HUMIMC101, and 7 × 10^3^ cell/cm^2^ for HUMIMC107. The following day, media was removed and mTeSRplus media was added for further 24 h. On the third day of culture, medium was changed to mTeSRplus medium supplemented with DMSO; none (negative control), 1%, or 2% v/v for 24 h.

### Pluripotency Cell Surface Markers by Flow Cytometry

Medium supplemented with DMSO was removed from hiPSCs cultured in 6-well plate after 24 h and wells were washed once with PBS. 1mL Accutase (STEMCELL Technologies) was added to each well and incubated at 37 °C for 10 min. Cell solution was gently pipetted to obtain a single cell suspension. A panel of fluorophore-conjugated pluripotency marker antibodies, TRA-1-81, TRA-1-60, SSEA3 and SSEA4 (BD Bioscience) was diluted in flow cytometry stain buffer (BD Bioscience) following suppliers’ guidelines to assess pluripotency (Table S1). An amount of 1 × 10^6^ cells were incubated with each antibody solution for 30 min in the dark at 4 °C. Cells were centrifuged at 300 g for 5 min and re-suspended in flow cytometry stain buffer. Flow cytometry was performed on BD Accuri C6 flow cytometer. Compensation parameters were assessed for each antibody and used to improve accuracy of final readout using FloJo analysis software.

### Pluripotency Intracellular Markers by Flow Cytometry

Medium supplemented with DMSO was removed from hiPSCs cultured in 6-well plate after 24 h and wells washed once with PBS. 1 mL Accutase (STEMCELL Technologies) was added to each well and incubated for 10 min at 37 °C. Cell solution was gently pipetted to obtain a single cell suspension. Accutase was deactivated with an equal part of DMEM medium and cells centrifuged at 300 g for 5 min. Supernatant removed and cell pellet re-suspended in 4% PFA and incubated for 30 min at 4 °C. Cells centrifuged at 300 g for 5 min and resuspended in permeabilization buffer (0.1% Triton X-100 in PBS) and incubated for 10 min at room temperature. Fluorescently conjugated antibodies (SOX2-PE and OCT3/4-AF647), were diluted in flow cytometry stain buffer (Table S1). 1 × 10^6^ cells were incubated with each antibody solution for 30 min in the dark at 4 °C. Cells were centrifuged at 300 g for 5 min and re-suspended in flow cytometry stain buffer. Flow cytometry was performed on BD Accuri C6 flow cytometer. Compensation parameters were assessed for each antibody and used to improve accuracy of final readout using FloJo analysis software.

### G1 Synchronisation Analysis

Medium supplemented with DMSO was removed from hiPSCs cultured in 6-well plate after 24 h and wells were washed once with PBS. 1 mL Accutase (STEMCELL Technologies) was added to each well and incubated at 37 °C for 10 min. Cell solution was gently pipetted to obtain a single cell suspension. Samples were centrifuged at 300 g for 5 min. Supernatant was removed and the cell pellet was re-suspended in 70% ethanol (diluted in MilliQ water). Cell suspension was incubated overnight at 4 °C followed by centrifugation at 300 g for 5 min. Supernatant was removed and the cell pellet was re-suspended in flow cytometry stain buffer supplemented with 50 µg/mL propidium iodide solution and 200 µg/mL RNAse and incubate for 3 h at 4 °C to stain DNA and remove RNA in each sample. Flow cytometry was performed on BD Accuri C6 flow cytometer. Data was analysed using FloJo analysis software.

### Kidney Organoid Differentiation

The protocol previously established by Morizane et al. for kidney organoid differentiation [21] was followed in this study. Firstly, basal media composed of Advanced RPMI (Gibco) and 1% GlutaMax (Gibco) was supplemented with CHIR99021 (Tocris) and 5ng/mL recombinant human noggin (Peprotech), was added to hiPSCs following the 24 h DMSO treatment previously described. Each different hiPSC line required a different concentration of CHIR99021; LUMC – 8µM CHIR99021, HUMIMC101–10µM CHIR99021, and HUMIMC107–12µM CHIR99021. Medium supplemented with CHIR99021 and noggin was refreshed after 2 days. On day 4, cell colony morphology was assessed. At this point colonies had condensed into tightly packed colonies with smooth edges. Medium composed of Advanced RPMI supplemented with 1% GlutaMax and 10ng/mL Activin A (Miltenyi Biotech), was added to cells for 3 days. On day 7 of differentiation, spent medium was removed and medium composed of Advanced RPMI supplemented with 1% GlutaMax and 10 ng/mL FGF9 (STEMCELL Technologies), was added to cells. By day 9 of the differentiation, vesicles were visible under brightfield microscopy indicating a successful MM NPC differentiation. Medium was refreshed every 2–3 days of culture. On day 14 of differentiation medium composed of Advanced RPMI supplemented with 1% GlutaMax was added to the culture and refreshed every 2–3 days. Kidney organoids with defined characteristic tubules visible under brightfield microscope were obtained on day 21 of culture. The number of individual organoid structures in each well of the 96 well plate was manually counted by eye.

### Generating LUMC-GFP + hiPSCs

For the genetic modification of the LUMC hiPSCs the super piggyBac transposase (Hera BioLabs) and LipofectamineTM Stem Cell Transfection Reagent (Invitrogen) were used following instructions from the manufacturers. To generate the LUMC-GFP + line, LUMC hiPSCs were passaged as single cells using Accutase on a Geltrex coated 24-well plate to a density of 75 × 10^3^ cells/well. Cells were maintained with mTeSR Plus media supplemented with 10 µM of Y-27,632 (STEMCELL Technologies) for 1 day. On the next day 50 µL of Opti-MEM media containing 1 µL of Lipofectamine, 500 ng of the PiggyBac plasmid and 500 ng of the plasmid of interest (pSH231-EF1-GFP-HYGRO) was prepared. pSH231-EF1-GFP-HYGRO was kindly provided by Raymond Monnat & Michael Phelps (Addgene plasmid # 115144; http://n2t.net/addgene:115144; RRID: Addgene_115144) [23]. This suspension was then added to each well containing the hiPSCs with fresh mTeSR Plus media and incubated overnight in cell culture conditions. The next day the media was refreshed to remove the dead cells and 200 µg/mL of Hygromycin (STEMCELL Technologies) for positive selection of the transfected cells. These cells were then further passaged as single cells to generate colonies that later were hand-picked for achieving a homogenous cell population. Transfected LUMC-GFP + cells were then assessed for pluripotency using SOX2 and OCT4 markers (Figure S2). hiPSC maintenance and differentiation was carried out following the same protocol as non-transfected LUMCs.

### Immunofluorescent Staining hiPSCs

All medium was removed from each well of 96-well plate and washed with PBS and cells fixed with 4% paraformaldehyde (Sigma) at 4 °C overnight. Samples were washed with PBS and stored for up to 4 months at 4 °C. Fixed plates were equilibrated at room temperature for 30 min to allow geltrex to fully crosslink before proceeding with staining protocol. Permeabilisation buffer composed of 0.1% v/v Triton-X (Sigma) was added to fixed cell culture for 10 min at room temperature. Blocking buffer composed by 3% w/v bovine serum albumin (BSA) (Sigma), was added to fixed cell culture for 1 h at room temperature. Primary antibody solutions were prepared by diluting antibodies/stains for specific proteins at desired concentration (Table S1) in 1.5% BSA and incubated with fixed samples overnight at 4 °C. Plates were removed from the fridge and allow to warm up to room temp for 30 min. Samples were washed three times with PBS at room temperature. Secondary antibody solutions were prepared by diluting fluorophore-conjugated antibodies including DAPI at desired concentration (Table S1) in 1.5% BSA and incubated with fixed samples for 1 h at 4 °C. Samples were washed three times with PBS at room temperature. All solutions made with 1X PBS were filtered through 0.2 μm syringe filter unit (Millipore) before the antibodies were added.

### Immunofluorescent Staining Nephron Progenitors and Kidney Organoids

All medium was removed from each well of 96-well plate and washed with PBS and cells/organoids were fixed with 4% paraformaldehyde (Sigma) at 4 °C overnight. Samples were washed with PBS and stored for up to 4 months at 4 °C. Fixed plates were allowed come to room temperature for 30 min, permeabilized with buffer composed of 0.1% v/v Triton-X (Sigma) for 15 min at room temperature. Blocking buffer composed by 0.3% v/v Triton-X, 0.05% Tween 20 (Sigma) and 3% bovine serum albumin (BSA) (Sigma), was added to fixed cell culture for 2 h at room temperature. Primary antibody solutions were prepared by diluting antibodies/stains for specific proteins at desired concentration (Table S1) in 1.5% BSA and incubated with fixed cell culture overnight at 4 °C. Plates were brough to room temp for 30 min and washed three times with permeabilisation buffer for 30 min. Secondary antibody solutions were prepared by diluting fluorophore-conjugated antibodies including DAPI at desired concentration (Table S1) in 1.5% BSA and incubated with fixed samples overnight at 4 °C. Plates were placed at room temp for 30 min and washed three times with permeabilisation buffer for 30 min.

### Image Acquisition and Processing

Images were acquired on automated inverted Nikon Ti-E microscope, equipped with a Lumencor Spectra Light source, an Andor Zyla 5.5 sCMOS camera, and an MCL NANO Z200-N TI z-stage, and the Leica TCS SP8 inverted laser scanning confocal microscope (Leica Microsystems). Live cell imaging was performed on an automated inverted Nikon Ti-E microscope, equipped with a Lumencor Spectra X Light source, Photometrics Prime 95B sCMOS camera, an MCL NANO Z500-N TI z-stage, and a Okolab incubator (37 °C, 5% CO_2_) (Fig. 2). Images were processed using NIS software (Nikon) and Fiji ImageJ.

### LDH Release Assay

LDH release was evaluated using the Invitrogen™ CyQUANT™ LDH Cytotoxicity Assay Kit (fluorescence-based), with alterations to manufacturer’s protocol outlined as follows. Cell-free culture medium was used to determine residual LDH in culture medium, while lysed cell control wells were used to represent maximum LDH release. Experimental conditions included untreated cells and treatment with 1% DMSO and 2% DMSO. Fluorescence was measured at Ex/Em = 560/590 nm using a plate reader, and LDH release was calculated according to a modified formula to that provided in the kit protocol, expressed as a percentage of LDH release normalized to residual LDH in culture medium and maximum controls. Maximum LDH activity and residual LDH medium were assessed in duplicate/triplicate wells, and the average values were used to calculate % LDH release for each compound treated well (i.e. wells treated with DMSO and negative control wells).

$$\begin{array}{c}\%\mathrm{LDH}\;\mathrm{release}=\\\left[\frac{\mathrm{compound}\;\mathrm{treated}\;\mathrm{LDH}\;\mathrm{activity}-\mathrm{average}\;\mathrm{residual}\;\mathrm{LDH}\;\mathrm{in}\;\mathrm{medium}}{\mathrm{average}\;\max\;\mathrm{LDH}\;\mathrm{activity}-\mathrm{acerage}\;\mathrm{residual}\;\mathrm{LDH}\;\mathrm{in}\;\mathrm{medium}}\right]\ast100\end{array}$$

### Oxidative Stress Assay

Oxidative stress was assessed using the CellROX^®^ Green Reagent (Invitrogen) following the manufacturer’s protocol with minor modifications. Cells were pre-treated with DMSO for 24 h. One hour prior to staining, a subset of cells was treated with 100 µM menadione as a positive control to induce oxidative stress. After the 1-hour induction, all samples (including positive controls and untreated controls) were incubated with CellROX^®^ Green Reagent at a final concentration of 5 µM for 30 min at 37 °C. Following incubation, the medium was removed, and cells were washed three times with 1X PBS to eliminate excess reagent. Cells were then fixed with 3.7% formaldehyde for 15 min at room temperature. Fluorescent imaging of the stained cells was performed using a 488 nm laser to detect ROS-associated green fluorescence.

### Quantitative PCR

All medium was removed from each well of 6-well plate wells. Plates were placed on ice and ice-cold PBS was added to each well for 5–10 min to dissolve Geltrex [24]. Cells were pipetted gently to resuspend, transferred to Eppendorfs and centrifuged at 750 g for 5 min at 4 °C. Cells were washed twice in ice cold PBS to remove all Geltrex and cell debris. The final cell pellet was dissociated and lysed using Trizol (Invitrogen) and stored at −80 °C until further use. Samples were thawed on ice and 20% v/v chloroform (Sigma) was added to lysed cell solution in trizol and centrifuged at 12,000 rpm for 15 min at 4 °C to separate phases. Upper aqueous phase containing RNA was transferred to a new Eppendorf tube and precipitated in isopropanol (Fisher BioReagents) for 2 h at −20 °C. RNA precipitates were centrifuged at 12,000 rpm for 30 min at 4 °C. RNA pellets were washed with ice-cold 75% v/v ethanol twice and centrifuged at 7500 rpm for 10 min at 4 °C. The final pellet was left to airdry for 10 min at room temperature and diluted in nuclease-free water (Qiagen). The RNA concentration was measured with the BioDrop µLITE device. RNA was stored at −80 °C until further use. The iScript cDNA sysnthesis kit (BioRad) was used to synthesize a quantitative amount of cDNA by reverse transcription. Briefly a mixture of 1 µg RNA template, 1 µL iScript reverse transcriptase, 1x iScript reaction mix, was prepared with nuclease free water up to 20 µL final volume. This solution was mixed on PeqStar thermal cycler (PeqLab) following iScript kit instructions (25 °C for 5 min, 46 °C for 20 min, 95 °C for 1 min, and finally, hold at 4 °C). Double stranded cDNA was stored at −20 °C. For qPCR, cDNA was added to the wells containing 0.5 µM validated gene specific forward primer and reverse primers (Table S2) and iQ SYBR green supermix (BioRad) to obtain a final volume of 10 µL. Plates were sealed and incubated on BioRad CFX96 following iQ SYBR green supermix kit instructions; (95 °C for 1 min) x1, (95 °C for 15 s, 60 °C for 45 s) x 39, (95 °C for 15 s) x1. All genes of interest were normalised against verified stable mitochondrial housekeeper gene nuclear encoded (ATP5PB).

### Single Cell Preparation for RNA Sequencing

LUMC hiPSCs cells untreated and treated with 1% and 2% DMSO for 24 h underwent dissociation using the Tissue Fixation & Dissociation for Chromium Fixed RNA Profiling protocol (CG000553, 10x Genomics). Cells were first washed twice with 1X PBS and dissociated using 1 ml of Accutase at 37 °C for 5–10 min. Detached cells were collected into conical tubes and the reaction was stopped by adding an equal volume of DMEM/F12. Following centrifugation at 300 rcf for 5 min, the supernatant was removed, and cells were resuspended in 1X cold PBS with 0.04% BSA and kept on ice. The suspension was filtered through a 40 μm cell strainer to remove aggregates, counted with a hemocytometer, and imaged to assess viability. Cells were stained with LIVE/DEAD Far Red stain (1 µl/ml) at 4 °C for 20 min in the dark, followed by washing with FACS buffer (1% BSA in PBS), and centrifugation at 300 rcf for 5 min at 4 °C. Paraformaldehyde was added to fixation buffer provided with the kit (3.7% final concentration) and incubated with cells at 4 °C for 22 h. After fixation, cells were washed and resuspended in 1X PBS with 1% ultra-pure BSA and RNase inhibitor (0.2 U/µl), imaged again, and incubated briefly in cold Quenching Buffer provided with the kit. FACS sorting was performed using a low-pressure setting and EDTA-/Mg²⁺-free sheath fluid. Sorted cells were collected in PBS with 1% BSA and RNase inhibitor, centrifuged (850 rcf, 5 min), and resuspended in cold Quenching Buffer. Viability and cell number were confirmed using trypan blue and a LUNA II automated cell counter. For storage, cells were mixed with Enhancer solution provided with the kit (100 µl) and 50% glycerol (275 µl per 1100 µl cell solution), gently pipetted, and transferred to −80 °C in a freezing container.

### Single Cell RNA Sequencing

Thawed fixed single-cell samples were processed using the Chromium Fixed RNA Profiling Reagent Kit for multiplexed samples protocol (CG000527, 10x Genomics). Samples were hybridized with human probe sets (BC001–BC008), loaded onto the 10x Genomics platform, and processed according to the manufacturer protocol. cDNA libraries were generated following the Chromium Fixed RNA Profiling Reagent Kits for Multiplexed Samples protocol (CG000527). Final cDNA library profiles were within the expected size range and shape, as verified by TapeStation analysis. No anomalies were observed during quality control. Constructed libraries were sequenced on the Illumina NovaSeq X Plus platform. FASTQ files were processed with *Cell Ranger* (10x Genomics, v 7.1.0) against GRCh38 to generate gene–barcode matrices, which were analysed in R v 4.4.1 using *Seurat* v 5.0.1. Matrices from the three conditions (untreated, 1% DMSO and 2% DMSO) were imported with Read10X, converted to individual Seurat objects and merged (add.cell.ids). Quality control removed cells with < 1 000 or > 10 000 detected genes or > 10% mitochondrial reads, retaining 26 007 cells and 18 082 genes. Data were log-normalised (scale factor 10 000), 2 000 highly variable features were selected with the *vst* method, and the matrix was scaled. Gene-set visualisations were produced with ggplot2 v3.5.1, patchwork v1.2.0 and the built-in Seurat plotting functions. All analysis scripts are available from the authors on request.

### SIX2 Protein Quantification by Flow Cytometry

Cells were detached from each well of 6-well plate using Accutase for 15 min at 37 °C, followed by washing and straining to remove debris and cell aggregates. Cells were counted, centrifuged, and PBS was removed. One-third of each condition was stained with fixable live/dead stain (1:1000 in PBS) in 50 µL for 30 min on ice, protected from Light. After adding 200 µL of FACS buffer (0.5% BSA in PBS), cells were centrifuged at 300 g for 3 min. The stain was removed, and cells were fixed with 2% PFA for 20 min at 4 °C. Cells were then washed with 300 µL FACS buffer and centrifuged again at 300 g for 3 min. The fixative was removed, and cells were stored in FACS buffer at 4 °C until ready to stain. For staining, cells were centrifuged at 500 g for 3 min, treated with 50 µL of 0.1% Triton X for 15 min on ice, and centrifuged again at 500 g for 3 min. Cells were then centrifuged and incubated with 50 µL primary antibody solution (SIX2, 1:500, Proteintech) for 30 min on ice. After adding 1000 µL of PBS, cells were centrifuged again and incubated with 50 µL secondary antibody solution (Donkey anti-rabbit AlexaFluor488, 1:2500, Invitrogen) in 1.5% serum solution for 15 min on ice. The cells were washed with 1000 µL PBS, centrifuged, and resuspended in 200 µL 0.5% BSA. Compensation beads (AbC™ Total, Invitrogen) were vortexed, and 10 µL of each sample (fluorophore only) was taken. Samples were incubated with 50 µL antibody solution for 30 min at room temperature in the dark, washed with 1000 µL FACS buffer, centrifuged, and resuspended in 200 µL 0.5% BSA. Each sample was then analysed using spectral flow cytometer Cytek Aurora (Cytek Biosciences), measuring AF 405 to detect dead cells (Live/Dead™ Fixable Aqua Dead Cell Stain Kit, ThermoFisher) to exclude them from analysis and AF488 to identify SIX2 + cell population. Unstained cells and live/dead stain cells were used to establish fluorescence background and define gates that would select single and live cells before analysing SIX2 stained samples. We analysed over 30 × 10^3^K cells per sample ensuring the Live cell count was above 10× 10^3^. Data analysis was performed using flow cytometer software, where percentage of SIX2 + population was given per at least 10 × 10^3^ live cells counted. Further analysis was performed using GraphPad Prism (see below).

### In-cell Western

Selected wells that contained kidney organoids, cultured in Greiner 96 well plate, were washed with PBS to remove all media and fixed with 4% paraformaldehyde (Sigma) at 4 °C overnight. Samples were washed with PBS and stored for up to 4 months at 4 °C. Fixed plates were allowed to equilibrate to room temperature for 30 min to allow geltrex to fully crosslink before proceeding with staining protocol. Permeabilisation buffer composed of 0.1% v/v Triton-X (Sigma) was added to fixed cell culture for 15 min gently rocking at room temperature. Blocking buffer composed of 0.3% v/v Triton-X, 0.05% Tween 20 (Sigma) and 3% bovine serum albumin (BSA) (Sigma), was added to fixed cell culture for 2 h gently rocking at room temperature. Primary antibody solutions were prepared by diluting antibodies/stains for specific proteins at desired concentration (Table S1) in 1.5% BSA and incubated with fixed cell culture for 24 h at 4 °C. Plates were equilibrated to room temp for 30 min and washed three times with permeabilisation buffer for 30 min at room temperature. Near infrared secondary antibody and CellTag700 total protein stain were added at desired concentration (Table S1) to 1.5% BSA and incubated with fixed cell culture overnight at 4 °C. After being placed at room temperature for 30 min, the samples were washed three times with 0.05% v/v tween-20 (Sigma) diluted in PBS for 15 min. LICOR Odyssey CLX was used to acquire near infrared (NIR) fluorescent signal at 4 mm offset height. Signal intensity was quantified using LiCOR Image Studio Ver 5.0 software. In each plate one well was incubated with each secondary antibody only and used to measure background signal. Total protein staining signal was used to normalize the staining signal for each of the proteins of interest.

### Statistical Analysis

Statistical analysis was performed using GraphPad Prism8 (version 8.2.0) software. One-way ANOVA with Tukey’s multiple comparisons was used for all qPCR results, flow cytometry, cell count and in-cell western protein quantification. A p-value smaller than 0.05 was considered statistically significant (denoted with *p < 0.05, **p < 0.01, ***p < 0.001, ****p < 0.0001). Results are shown as mean ± standard error of mean.

## Results

### Pluripotency and Differentiation Potential

**Fig. 1:**
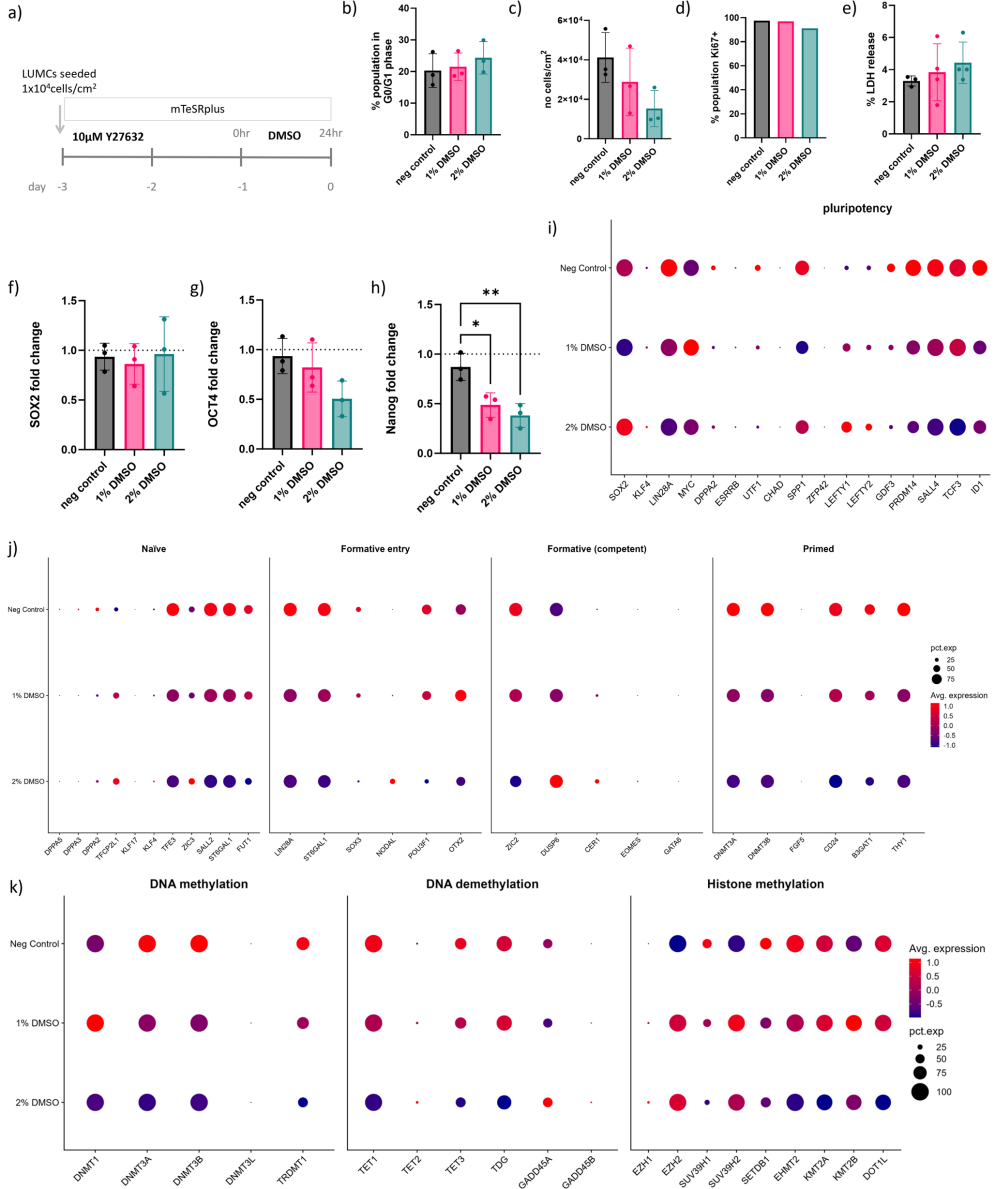
LUMC hiPSC pluripotency marker expression and cell cycle analysis following DMSO treatement: (**a**) Schematic of first 3 days of hiPSC culture including 24 h DMSO treatment before commencing differentiation protocol. Characterization and quantifications performed at endpoint - day 0: (**b**) The percentage of cells in G0/G1 phase, and (**c**) the number of hiPSCs counted/cm^2^ (*N* = 3). (**d**) The percentage hiPSCs expressing proliferation marker Ki67 (*N* = 1). (**e**) Percentage LDH release in the culture medium (*n* = 4). Gene expression analysis by qPCR of transcription factors for pluripotency; (**f**) SOX2, (**g**) OCT4 and (**h**) NANOG (*N* = 4). RNA-seq dot plots showing the expression levels and percentage of cells expressing genes associated with; (**i**) pluripotency, (**j**) states of pluripotency, and (**k**) DNA methylation, DNA demethylation and histone methylation (*N* = 1)

To investigate the effect of DMSO on the LUMC hiPSCs Line used, quantitative and qualitative assessment of key markers was performed three days post-seeding or day 0 of the differentiation protocol. (Fig. 1a).

Following three-day culture on plates coated with basement membrane extract (BME), Geltrex, including a 24 h treatment with 1% or 2% DMSO, and an untreated negative control we observed a slight increasing trend in the number of cells in the G1 phase correlated with an increase in DMSO concentration used. The percentage of cells in the G0/G1 phase was quantified (Fig. 1b) by distinguishing % cell population with a single copy of DNA to determine the G0/G1 phase of the cell cycle (Figure S1a). We found that there was a substantial decrease in the overall number of cells with DMSO treatment (Fig. 1c). To exclude the possibility of DMSO affecting proliferation, we assessed if the cells remaining in colonies were still proliferating. Immunofluorescent images of hiPSC colonies depicted localization of Ki67 protein expression, a marker of cell proliferation. Ki67 expression was assessed (Figure S1b). Flow cytometry data showed a non-significant decrease in the percentage of cells expressing Ki67 with the increase of DMSO (Fig. 1d).

To evaluate if cell death was contributing to reduction in cell number, we assessed LDH release after 24 h DMSO treatment and found an increase of LDH levels in wells treated with DMSO (Fig. 1e). ROS activity was also assessed using CellROX assay (Invitrogen) with no major differences distinguished between different conditions (Figure S2a). Gene expression levels of heat shock protein were assessed by qPCR (Figure S2b-c. HSP5a was found to be upregulated in hiPSCs treated with 2% DMSO, whereas HSP90B1 showed no difference between different conditions. To assess broader transcriptional changes, we performed single-cell RNA sequencing (scRNA-seq) following DMSO treatment. scRNA-seq data revealed transcriptomic modulations in apoptosis related genes following DMSO treatment (Figure S2d).

Pluripotency was assessed following DMSO treatment by measuring gene expression of transcription factors SOX2, c-Myc, OCT4 and NANOG by qPCR analysis (Fig. 1f-i). SOX2 and c-Myc remained stable (Fig. 1f-g). Interestingly, OCT4 expression decreased slightly (Figure h) and expression of the non-critical pluripotent transcription factor, NANOG, decreased significantly (Fig. 1i). Pluripotency was also assessed following DMSO treatment using a standard panel of antibodies for pluripotency transcription factors and surface protein markers which confirmed pluripotency was maintained in all conditions (Figure S3a-e). In order to evaluate if DMSO was causing early onset differentiation we assessed a panel of genes associated with each of the three germ layers but found that low expression levels and no evidence of a significant shift towards any of the three germ layers (Figure S3f).

Further analysis of the scRNA-seq data revealed additional gene expression alterations that support our previous findings (Fig. 1j), including downregulation of CCND1/2/3. The degradation of these cyclins is known to be associated with G1 phase arrest. Furthermore, we observed upregulation of cell cycle inhibitor p27 (CDKN1B), suggesting that DMSO does influence cell cycle dynamics. Next, the scRNA-seq data was processed to specifically examine genes known to be associated with distinct pluripotency states; naïve, formative, and primed (Figure S1c). This analysis revealed a shift in transcriptomic profile following DMSO treatment. Notably, several genes characteristic of the primed state were downregulated, including DNMT3A and DNMT3B, which are key regulators of DNA methylation. Given that DMSO has been previously shown to influence DNA methylation and chromatin accessibility, we extended our analysis to include genes involved in epigenetic regulation, including DNA methylation (DNMT3A, DNMT3B), DNA demethylation (TET enzymes), and histone methylation modifiers (Fig. 1k). We observed a consistent downregulation of genes in these categories, suggesting that DMSO treatment may broadly suppress methylation capacity and reconfigure the epigenetic landscape during early differentiation. These findings may reflect a shift in the transcriptional and chromatin environment that supports enhanced lineage competence in hiPSCs prior to commencing kidney organoid differentiation culture.

### Changes in hiPSC Colony Morphology Following DMSO Treatment

**Fig. 2:**
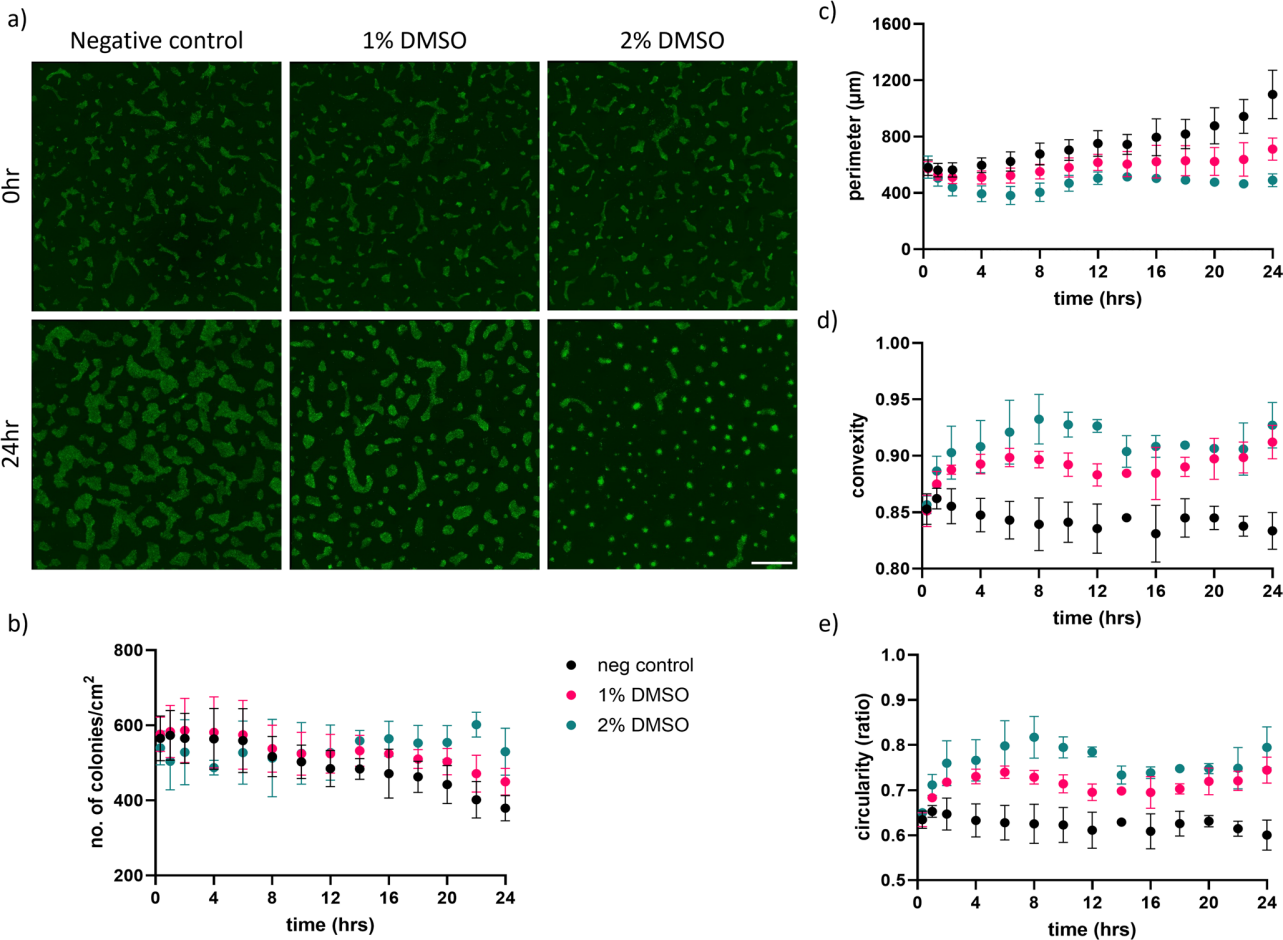
Parametric analysis of LUMC-GFP+ hiPSC colony morphology over 24hr DMSO treatment. (**a**) Fluorescence images of LUMC-GFP+ hiPSC colonies at the beginning (0 hr, upper panel) and end (24 hrs, lower panel) of the exposure to DMSO. Scale bar of 1000 μm. Real-time measurements of (**b**) number of colonies/cm^2^, (**c**) perimeter, (**d**) convexity and (**e**) circularity over 24hr DMSO treatment (*N*=3)

The impact of cell morphology was analysed upon DMSO exposure. Results shown in Fig. 2 provide a parametric analysis of hiPSC colony morphology at the beginning (0 h) and at the end (24 h) of the exposure to DMSO (Fig. 2a). The LUMC-GFP + hiPSCs pluripotency OCT4 and SOX2 (Figure S4a) was confirmed and with levels comparable to non-GFP LUMC line.

Fluorescence images of LUMC-GFP + colonies were taken at 20 min intervals over the course of the 24 h DMSO treatment to identify the morphological changes in real-time (video S1-S3). A comparative analysis was performed on images acquired throughout 24 h DMSO treatment (Fig. 2b-e) using consistent settings on NIS software. The number of colonies was quantified in each image. The DMSO treatment resulted in an increase in the overall number of colonies for the start of differentiation as fewer colonies merged in wells treated with 1–2% DMSO compared to the non-treated control over the course of 24 h (Fig. 2b, S5a, Video S1a-c). This behavior was also observed for LUMCs imaged under brightfield (Video S2a-c). Quantification of fluorescent imaging of LUMC-GFP⁺ colonies revealed that 24-hour DMSO treatment led to a decrease in colony perimeter with increasing DMSO concentration (Fig. 2c, S5b), alongside an increase in colony convexity (Fig. 2d) and circularity (Fig. 2e, S5c), a known morphological feature of pluripotent stem cell colonies. At 24 h timepoint there was an overall statistical difference in all measurements between different conditions (Figure S5a-c). These measurements offered insights that clear morphological alterations to hiPSC colonies were observed following the DMSO treatment, which varied with the concentrations tested.

### HiPSC Structural Rearrangement and Integrin Binding

**Fig. 3:**
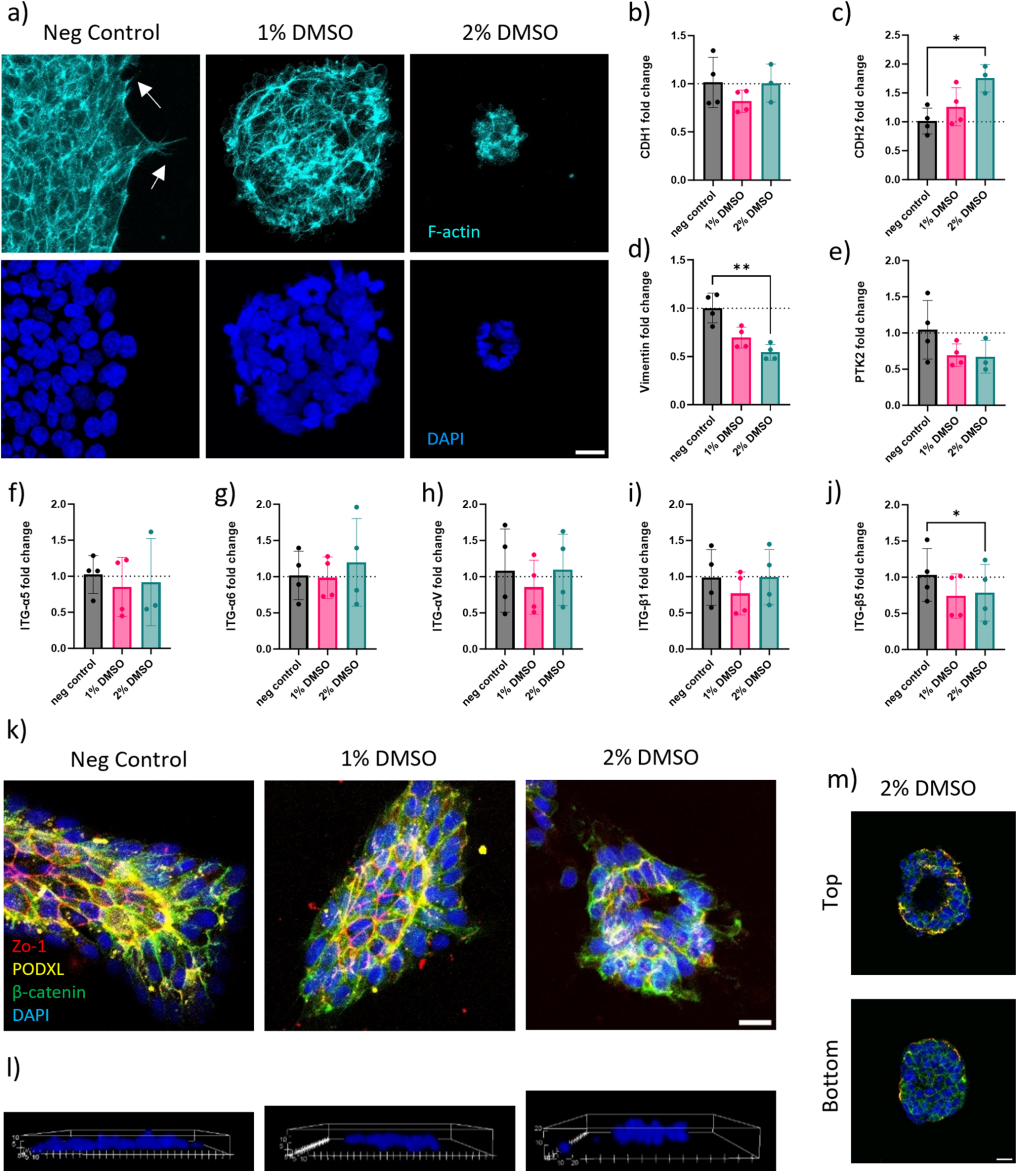
Changes in LUMC hiPSC colony structural organisation following DMSO treatment. (**a**) Immunofluorescence images of F-actin cytoskeleton stained with Phalloidin (cyan) and DAPI (bue). Cellular protrusion indicative of focal adhesions indicated with white arrows. Gene expression analysis of (**b**) E-cadherin (CDH1), (**c**) N-Cadherin (CDH2), and (**d**) Vimentin. Gene expression analysis of (**e**) focal adhesion-associated protein kinase (PTK2), and (**f**) integrin alpha-5 (ITG-α5), (**g**) integrin alpha-6 (ITG-α6), (**h**) integrin alpha-V (ITG-αV), (**i**) integrin beta-1 (ITG- β1), (**j**) integrin beta-5 (ITG- β5) (*N* = 4). (**k**) Immunofluorescence images of hiPSC colonies stained for β-catenin (green), ZO-1 (red), and podocalyxin (yellow) acquired on confocal and shown as 2D maximum intensity projection of an acquired z-stack and (**l**) 3D rendered images of a representative colony showing height of colonies in z-plane. (**m**) Single stack image from Z-stack from upper section (top) and lower section (bottom) of a representative hiPSC colony treated with 2% DMSO. Scale bar of 20 μm applicable to all images

With the changes previously reported on the colony morphology, a deeper investigation was performed on hiPSC cytoskeletal organization, as well as gene expression analysis related to integrins, and cell adhesion molecules (Fig. 3).

Immunofluorescence images of the F-actin cytoskeleton, stained with Phalloidin (cyan), highlighted a gradual change at the cellular level and in the structural organisation of the hiPSC colonies (Fig. 3a). Notably, there was a lack of pronounced cell protrusions, indicative of focal adhesions, on the periphery of the colonies exposed to DMSO when compared to non-treated colonies. Hence, the gene expression of critical components involved in integrin-mediated signalling and focal adhesions in stem cells were further analysed. Gene expression analysis revealed no change in E-Cadherin (CDH1) (Fig. 3b), and upregulation of cell-cell adhesion molecule N-cadherin (CDH2) (Fig. 3c) following the DMSO treatment. Focal adhesion kinase (PTK2), known to interact with integrin-mediated adhesion complexes and Vimentin were also downregulated with DMSO treatment (Fig. 3d, e). The integrins (α5, α6, αν, β1, and β5), abundantly expressed on hiPSC and known to play important roles in binding induced hiPSCs to basement membrane components including collagen, fibronectin, laminin, and vitronectin were also analysed. The gene expression of most integrins remained unchanged with exception to both β1 and β5 integrins which was downregulated with DMSO treatment (Fig. 3f-j).

We also observed morphological changes in colonies present in DMSO treated cultures, although more abundantly in 2% DMSO culture; small, condensed, circular colonies were observed (Figure S3a). These small colonies show lumen forming in the center (Fig. 3m). Additionally, the lumen-forming protein podocalyxin was expressed at the top and centre of each colony (Fig. 3k, m). These findings collectively suggest that DMSO treatment significantly influences the cytoskeletal organization of hiPSCs and gene expression of integrin, cell adhesion molecules and adhesion sites on the periphery of cell colonies.

### Inducing Mesoderm Induction Following DMSO Treatment of hiPSCs

**Fig. 4:**
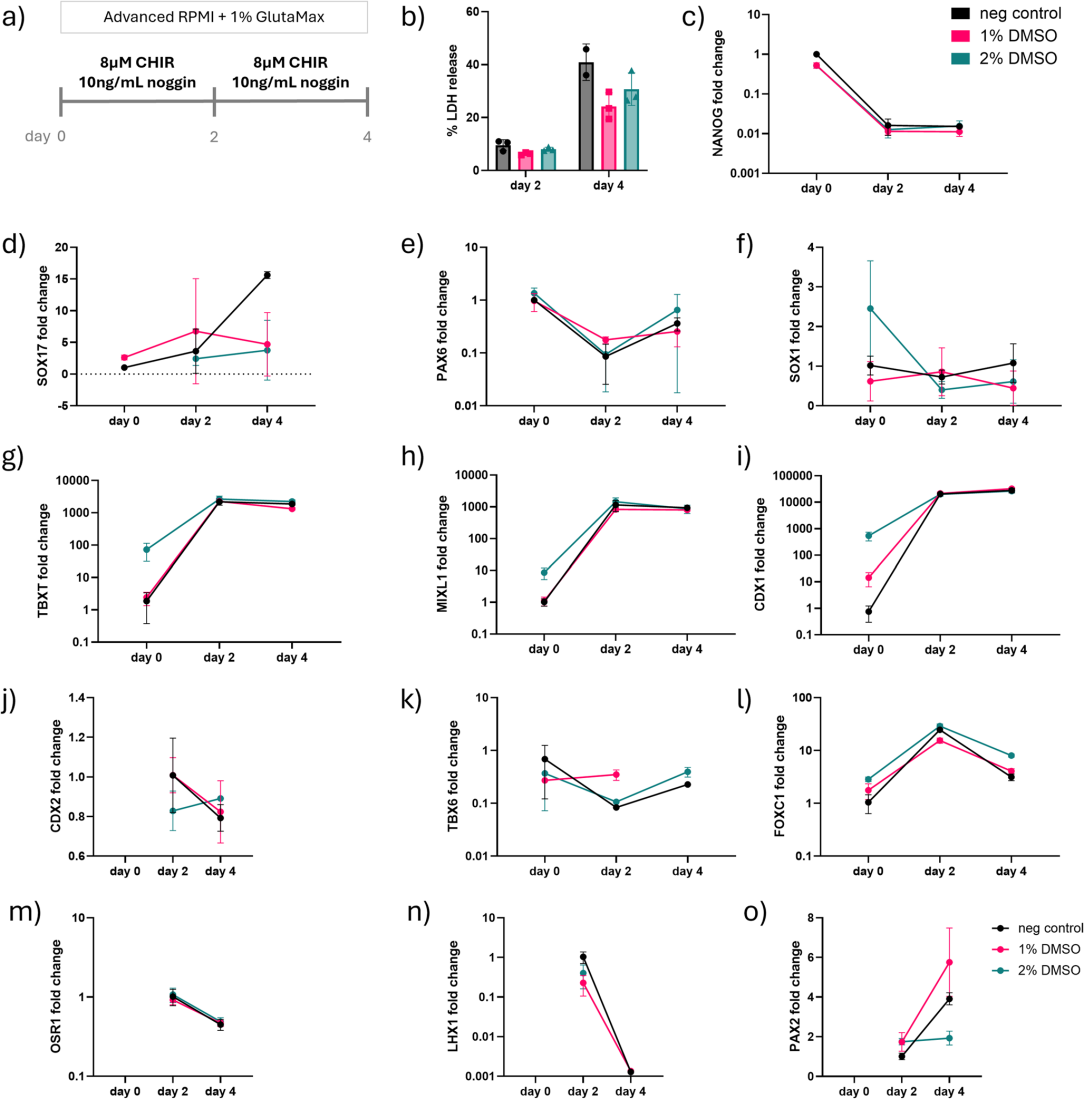
Gene expression analysis during LUMC hiPSC mesoderm induction (**a**) Schematic of the first 4 days of kidney organoid differentiation with Wnt agonist (CHIR) and BMP4 inhibitor (noggin) following 24 h DMSO treatment. (**b**) Percentage LDH release in the culture medium on day 2 and 4 of kidney organoid differentiation (*n* = 3). Gene expression analysis of pluripotency marker (**c**) NANOG, endoderm marker, (**d**) SOX17, ectoderm markers, (**e**) PAX6 and (f) SOX1, primitive streak markers, (**g**) TBXT and (h) MIXL1, posterior primitive streak markers, (**i**) CDX1 and (**j**) CDX2, paraxial mesoderm markers (**k**) TBX6 and (**l**) FOXC1, and intermediate mesoderm markers (**m**) OSR1, (**n**) LHX1 and (**o**) PAX2 (*n* = 3). Omission of data points in the gene expression panels indicates undetectable or too low to quantify expression levels

The first stages of kidney organoid differentiation require treatment with Wnt agonist (CHIR99021) and BMP inhibitor (Noggin). At this stage, we assessed if the pre-treatment of hiPSCs with DMSO affected cell viability following DMSO treatment during the first 4 days of kidney organoid differentiation (Fig. 4a).

Previously, we mentioned that DMSO treatment results in increased cytotoxicity; however, we found that cells treated with DMSO showed reduced % LDH release on day 2 of differentiation and further reduction on day 4 (Fig. 4b). The Wnt agonist is used to induce primitive streak, and the BMP inhibitor prevents induction into posterior primitive streak. We therefore wanted to compare how these early stages of differentiation were affected by DMSO treatment of LUMC hiPSCs before commencing differentiation. Results showed that the genes for pluripotency, such as NANOG, were downregulated with Wnt agonist induction for all conditions (Fig. 4c). Next, we assessed the level of characteristic Lineage markers for the three germ layers during the first 4 days of kidney organoid differentiation. We found that the endoderm marker SOX17 was upregulated for the negative control, whereas DMSO pre-treated cells showed no upregulation (Fig. 4d). Characteristic markers of the ectoderm lineage, PAX6 and SOX1, were generally unchanged in all conditions (Fig. 4e-f).

To further understand the impact of DMSO pre-treatment on early kidney organoid differentiation dynamics, we examined the expression of primitive streak markers, as this stage is critical for initiating mesodermal commitment during kidney organoid development (Figure S6a). We observed that both TBXT and MIXL1 were upregulated during the early phase of differentiation, indicating successful induction of the primitive streak (Fig. 4g-h). We also assessed posterior primitive streak markers. CDX1 showed clear upregulation on day 2 of differentiation in all conditions (Fig. 4i), while CDX2 expression emerged on day 2 but was subsequently downregulated by day 4 in both the negative control and 1% DMSO conditions (Fig. 4j). Interestingly, expression of TBX6, a marker associated with paraxial mesoderm (Fig. 4k), remained largely unchanged across conditions. We also assessed FOXC1, a paraxial mesoderm marker (Fig. 4l), which showed an increase in expression from day 0 to day 2, followed by downregulation by day 4 across all conditions. Intermediate mesoderm markers OSR1 and LHX1 were detected on day2, although expression levels remained low across all conditions (Fig. 4m-n). In contrast, PAX2 expression began to increase by day 4 specifically in the 1% DMSO pre-treated condition (Fig. 4o). This may indicate a slight acceleration in intermediate mesoderm specification in response to DMSO pre-treatment.

To evaluate progression past intermediate mesoderm fate, we examined markers expected to emerge during this stage of differentiation. Expression of HOXD11, a posterior intermediate mesoderm marker, was upregulated in 2%DMSO on day 0 of differentiation followed by downregulation to the same as other conditions by day 2 and no change on day 4 (Figure S6b). Metanephric mesenchyme marker, Wt1 remained low from day 0 to day 4 of differentiation (Figure S6c). We also evaluated the expression of NKX2.5, a marker of early cardiac mesoderm, to assess potential off-target differentiation. We found a transient increase in NKX2.5 expression between day 0 and day 2, which was subsequently downregulated from day 2 to day 4 in the 1% DMSO condition (Figure S6d). This suggests that while early off-target lineage priming may occur, it is not sustained through the differentiation process in DMSO pre-treated cells.

### Metanephric Mesenchyme Nephron Progenitor Differentiation

**Fig. 5:**
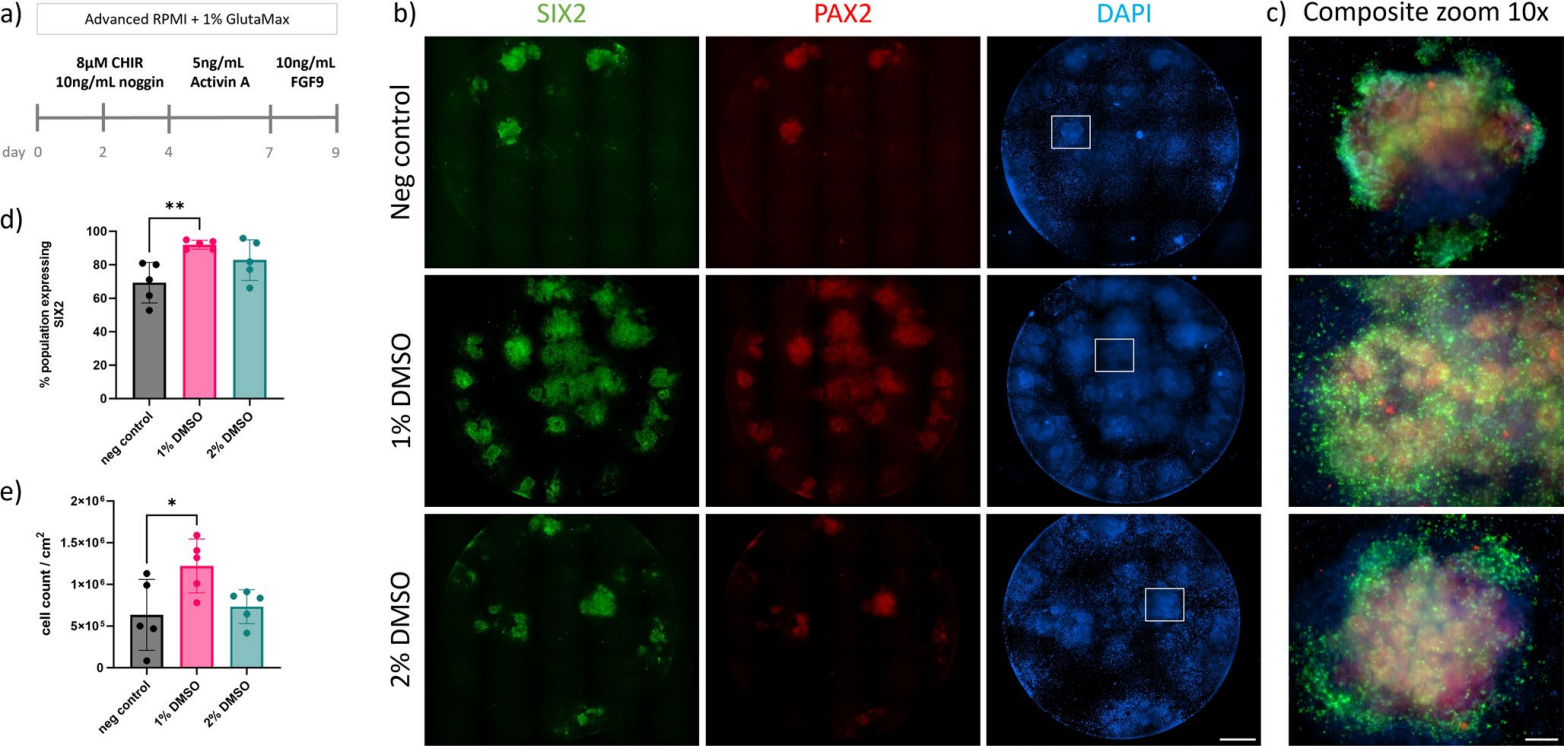
LUMC hiPSC-derived metanephric mesenchyme nephron progenitor cells on day 9 of kidney organoid differentiation. (**a**) Schematic of the first 9 days of kidney organoid differentiation protocol up to the stage when nephron progenitors emerge. (**b**) Immunofluorescence images of differentiated hiPSCs stained for MM markers; SIX2 (green) and PAX2 (red). Scale bar – 1000 μm. (**c**) Composite zoom 10x images, scale bar – 100 μm. (**d**) Percentage of SIX2 + cells at day 9, measured by flow cytometry (*N* = 5). (**e**) The number of cells/cm^2^ counted following dissociation using a manual haemocytometer (*N* = 5)

The main aim of this study was to investigate the effects of DMSO treatment on hiPSC in differentiation efficiency, specifically in becoming nephron progenitors (NPCs), therefore we investigated the induction of critical NPC markers PAX2 and SIX2 expression (Fig. 5).

Cells were analysed at day 9 of the kidney organoid differentiation protocol, as depicted following the developmental timeline up to the stage when nephron progenitors emerge (Fig. 5a). Immunofluorescence images showed hiPSCs successfully differentiated towards nephron progenitors presenting abundant expression of MM markers SIX2 (green) and PAX2 (red), when treated with 1% DMSO (Fig. 5b, c). The percentage of MM nephron progenitors represented by SIX2 + cells in culture was quantitatively assessed using flow cytometry, providing insight into the efficiency of differentiation. The population of SIX2 + cells in non-treated hiPSCs reached 69%, treated with 1% DMSO reached on average 92%, and treated with 2% DMSO reached 83% (Fig. 5d, S7). Additionally, the number of cells per cm² following dissociation was manually counted using a haemocytometer. Non-treated hiPSCs resulted on average in 6 × 10^5^cells/cm^2^, treated with 1% DMSO results in 12 × 10^5^ cells/cm^2^, and treated with 2% DMSO resulted in 7 × 10^5^ cells/cm^2^ (Fig. 5e). The assessment of the MM progenitor differentiation was also confirmed with the LUMC-GFP + expressing Line, on day 9 of the kidney organoid differentiation and these also expressed the markers for nephron progenitors SIX2 and PAX2 (Figure S4b);

This analysis underscores the successful differentiation of hiPSCs into nephron progenitors, where treatment of hiPSC with 1% DMSO leads to highest number of renal progenitors (identified as SIX2 + cells).

### Kidney Organoid Differentiation

**Fig. 6:**
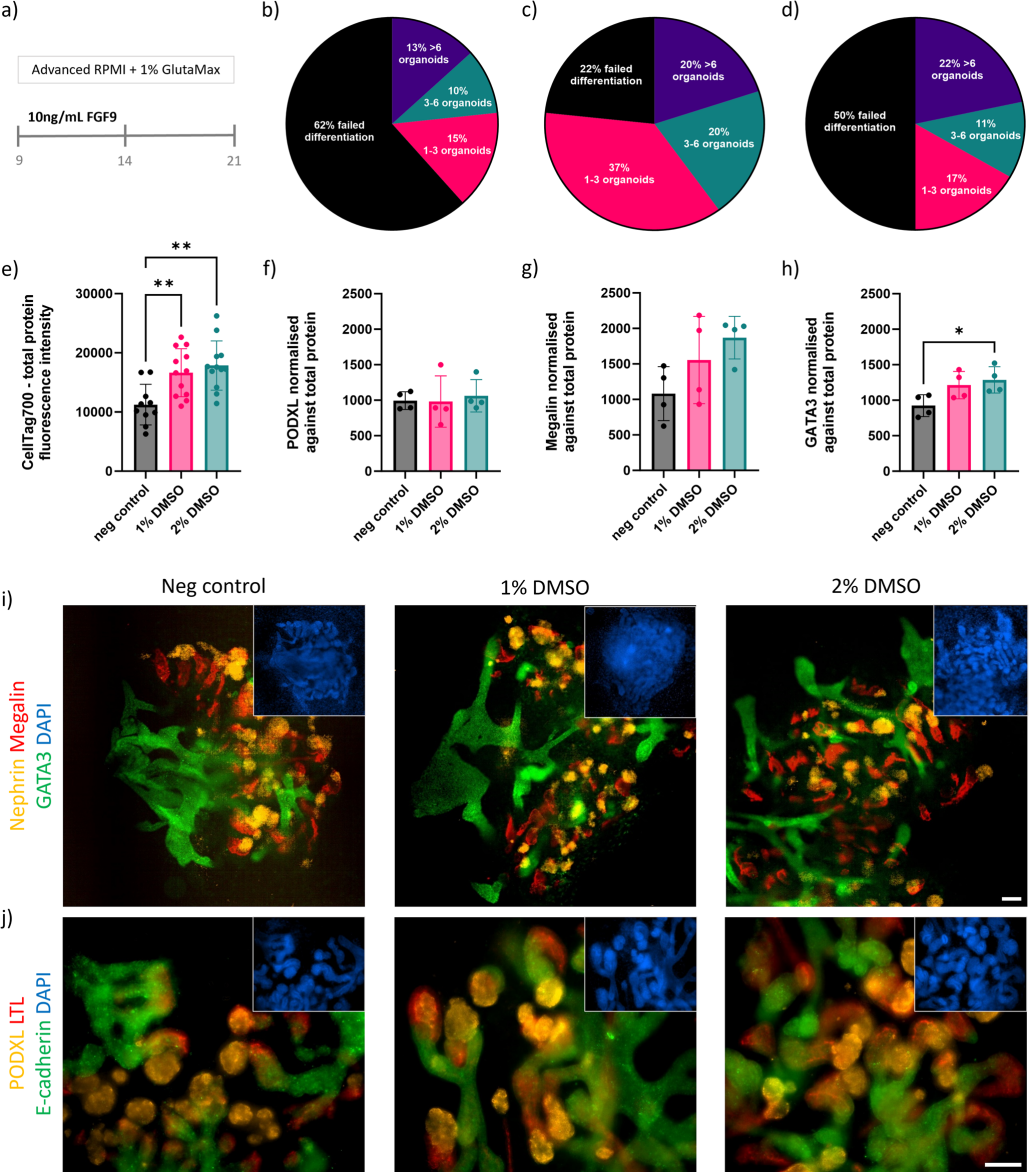
Characteristic LUMC hiPSC-derived kidney organoid protein expression images and differentiation efficiency quantification on day 21 of the differentiation protocol. (**a**) Schematic of the final 12 days of differentiation from emergence of nephron progenitors on day 9 to kidney organoids on day 21. b-d) Percentage of wells (*n*=60) with successfully differentiated kidney organoid colonies developing; (**b)** no treatment, (**c)** 1% DMSO, (**d)** – 2% DMSO. (**e**) Total fluorescent signal measured in each well (*n* = 5), and used to calculate normalised signal of characteristic proteins found in different sections of the developing nephron; (**f**) podocalyxin – glomerulus, (**g**) megalin - proximal tubule, (**h**) and GATA3 - distal tubule/collecting duct. Representative immunofluorescence images of day 21 kidney organoids showing a general overview of each well with characteristic proteins found in different sections of the developing nephron: (**i**) nephrin – glomerulus (yellow), megalin - proximal tubule (red), and GATA3 - distal tubule and collecting duct (green). (**j**) podocalyxin – glomerulus (yellow), lotus tetragonolobus lectin (LTL) – proximal tubule (green), and E-cadherin – distal tubule (red). Scalebar of 100 μm valid for all images

To assess if the DMSO treatment affected nephrogenesis, the differentiation culture was continued in 2D setting until day 21. Cell culture wells were accessed on the final day of the kidney organoid differentiation protocol (Fig. 6).

A schematic outlines the final 12 days of metanephric mesoderm differentiation of NPC culture toward kidney organoids (Fig. 6a). The number of individual kidney organoids identified as characteristic tubular structures developing in each well of a 96-well plate was manually counted for each condition (Figure S8a). The percentage of wells with successfully differentiated kidney organoids for each condition is represented in Fig. 6b-d. The results showed that 38% of the wells containing cells not treated with DMSO contained kidney organoids with characteristic tubular structures, while 78% and 50% of wells contained kidney organoids for cells treated with 1% and 2% DMSO, respectively. A similar trend was observed under brightfield in 6 well plate format (Figure S8b).

The fluorescent signal for key proteins characteristic of different nephron sections was measured and normalised against total protein (Figure S9). Total protein content results (Fig. 6e) showed the protein signal correlated with the number of kidney organoid structures present. Overall, the amount of podocalyxin (glomerulus) signal did not vary across conditions (Fig. 6f). The signal for megalin (proximal tubule) increased in wells treated with higher concentraion DMSO (Fig. 6g). A significant increase was measured for signal of GATA3 (distal tubule/collecting duct) in wells treated with 2% DMSO compared to non-treated control (Fig. 6h). Immunofluorescent images of kidney organoids showed the structural arrangement of the developing nephrons for each condition and expression of characteristic proteins found in the different segments of the nephron. The kidney organoids differentiated in each condition showed expression of Podocalyxin (PODXL) for the podocytes in the glomerulus, lotus tetragonolobus lectin (LTL) for brush boarder of the proximal tubule, and E-cadherin located in the epithelium of the developing distal tubule and collecting duct (Fig. 6i, S8c), as well as nephrin located at the slit diaphragm of the glomerulus, functional transporter protein megalin in the proximal tubule, and GATA3 located in the epithelium of the distal tubule and collecting duct (Fig. 6j, S8c) in each of the respective tubular structures. The assessment of the kidney organoid differentiation was also confirmed with the H101 and H107 hiPSC Lines, on day 21 of the kidney organoid differentiation and these also expressed the markers for kidney organoid markers, GATA3, PODXL and Megalin (Figure S5e). The LUMC-GFP + line on day 21 of kidney organoid differentiation also showed that the tubular structures emerging expressed nephron markers, Megalin and GATA3 (Figure S5e). Furthermore, the degree of successful differentiation was again observed for the different hiPSCs Lines investigated, with a clear advantage when 1% DMSO was used (Figure S5d).

This analysis highlights the impact exposure of hiPSCs to DMSO has on the downstream differentiation of kidney organoids. The protein quantification presented some significant changes only in the expression of, the distal tubule/collecting duct marker GATA3, between tested conditions. We also found the treatment with 1% DMSO presented the highest rate of organoid formation.

## Discussion

### Effects of DMSO on hiPSC Pluripotency, Proliferation, and Epigenetic Regulation

This study aimed to investigate how 1–2% DMSO treatment for 24 h affects hiPSC differentiation. We performed a detailed phenotypic and transcriptomic analysis using hiPSCs to better understand how 24 h low dose DMSO treatment influences pluripotency and differentiation readiness. Quantitative and qualitative assessments were conducted three days post-seeding, coinciding with the start of differentiation revealing changes associated with cell cycle, proliferation, viability, and pluripotency.

Consistent with previous research on use of DMSO for hiPSC culture we observed a dose-dependent increase in the proportion of cells in the G1 phase following DMSO exposure, as determined by DNA content analysis [18]. This finding aligns with the observed downregulation of cyclins: CCND1/2/3, and CDC25A from our scRNA-seq dataset, which are known to regulate G1 to S-phase progression [25–27]. Despite the reduction in overall cell numbers, Ki67 expression analysis indicated that the remaining cells within colonies continued to proliferate, suggesting that DMSO does not fully suppress cell cycle activity but may contribute to cell cycle arrest in the G1 phase.

Given the observed reduction in cell number, we evaluated potential cytotoxic effects of DMSO. Increased LDH release in DMSO-treated wells indicated membrane damage and cell death, while ROS levels remained unchanged, suggesting oxidative stress was not a major contributor. Notably, expression of the heat shock protein HSPA5 was upregulated at the transcript level in response to 2% DMSO, indicating a mild cellular stress response [28]. Additionally, scRNA-seq data showed modulation of apoptosis-related genes, supporting the involvement of stress or cell death pathways. The modulation of apoptosis genes and their interplay with Wnt signalling may explain why we observe less cell death in DMSO treated hiPSCs in the first few days of differentiation culture [29].

To determine whether DMSO treatment affects the maintenance of pluripotency in hiPSCs, we evaluated the expression of key transcription factors and surface markers associated with the pluripotent state. Protein-level expression of OCT4 and SOX2 remained evident across colonies in all conditions, although flow cytometry revealed a modest but significant reduction in these proteins at higher DMSO concentrations. Importantly, pluripotency surface marker expression (SSEA3, SSEA4, TRA-1-60, and TRA-1-81) remained high, and immunostaining confirmed retention of pluripotency-associated features across conditions [30, 31]. Our results showed that DMSO treatment did not significantly alter the protein expression of core pluripotency markers, suggesting that the pluripotent state was maintained at this level of analysis [17]. Transcriptionally, SOX2 and c-MYC levels remained stable, while OCT4 and NANOG were reduced, with NANOG showing the most significant decrease. NANOG is associated with the high proliferation rate characteristic of pluripotent stem cells. Previous studies have shown that NANOG is linked to the progression from the G1 to S phase of the cell cycle via regulation of the cell cycle inhibitor p27 (CDKN1B) [32, 33]. We show that CDKN1B is upregulated with increasing DMSO concentration. Therefore, our findings align with the known effects of DMSO on the cell cycle, where DMSO has been reported to increase the population of pluripotent stem cells in the G1 phase through downregulation of genes associated with cell cycle regulation RNAseq [34, 35].

During transition from formative to primed state, NANOG is often downregulated [36, 37]. To explore this further, we examined genes associated with naive, formative, and primed pluripotency states in our scRNA-seq dataset [38]. DMSO treatment resulted in a transcriptomic shift marked by downregulation of several primed-state genes, including DNMT3A and DNMT3B, which are also key enzymes in DNA methylation [39]. Broad suppression of genes involved in DNA methylation, demethylation (TETs), and histone methylation suggests that DMSO alters the epigenetic regulatory landscape [40, 41].

Taken together, these findings indicate that short-term DMSO treatment at low concentrations does not compromise pluripotency, but instead primes cells by modulating the cell cycle, inducing mild cellular stress, and reshaping transcriptional and epigenetic networks. These changes may enhance lineage competence and responsiveness to subsequent differentiation cues, particularly in kidney organoid protocols that rely on precise timing and epigenetic plasticity.

### Changes in hiPSC Colony Morphology

DMSO-treated hiPSCs exhibited distinct morphological changes, prompting us to measure these alterations and correlate them with established knowledge about the relationship between hiPSC colony structure and pluripotency. Previous research has shown that compact colonies with tight cellular associations are characteristic of successful hiPSC conversion [1], and smaller colonies tend to have higher differentiation potential due to the greater proportion of cells at the colony edges [42]. This is further amplified by the increased number of individual colonies present in cultures treated with 1–2% DMSO.

Our results showed that colonies treated with DMSO presented a reduced perimeter, suggesting they are better candidates for differentiation. Additionally, DMSO treatment led to increased colony convexity and circularity, indicating less variability in colony morphology indicative of more stable hiPSCs [43]. Phalloidin staining revealed a more rounded cytoskeletal organization with fewer thin actin stress fibers at the colony edges, indicating possible alterations in mechanical forces and biophysical interactions within the colonies, potentially influencing their state of pluripotency and differentiation potential [44].

A notable observation was the emergence of small, round colonies with a lumen forming in the centre following 2% DMSO treatment consistent with previous reports [45]. We noticed this particular shape resembled epiblast spheroids [46]. These spheroids are reported to be primed for differentiation, particularly toward the mesoderm lineage [47]. They are characterised by formation of lumens with apicobasal polarity markers like PODXL, ZO-1, and β-catenin. Our study observed similar morphological features in DMSO-treated hiPSCs, suggesting that DMSO may induce a transition towards more “primed state” of pluripotency, similar to epiblast spheroids.

### HiPSC Structural Rearrangement and Interaction with BME

Cell protrusions indicative of focal adhesions on the periphery of hiPSC colony edges appear to be affected by DMSO treatment, leading us to investigate the effects on integrin expression and focal adhesion dynamics. Integrins, particularly α5, α6, αv, β1, and β5, play a crucial role in stem cell-matrix interactions and significantly influence human embryonic stem cell survival and differentiation [48]. Our analysis revealed that hiPSCs grown on BME, Geltrex - an extra-cellular matrix derived from Engelbreth-Holm-Swarm mouse sarcomas, expressed high levels of these integrins, consistent with previous reports. Following DMSO treatment, integrin expression levels remained stable, with a notable decrease in integrin β5 expression, potentially impacting cell attachment and proliferation [49]. Integrin interaction with extra-cellular matrix substrate activates focal adhesion kinase (FAK) and AKT signalling, which are critical for suppressing cytoskeletal contraction and preventing apoptosis [50, 51]. DMSO-treated hiPSCs showed slight downregulation of FAK expression, along with a loss of adhesion sites and a decrease in cell number, suggesting potential effects on cell proliferation or survival [17]. These findings highlight the impact DMSO treatment has on hiPSC cell attachment and interaction with BME, which can explain the reason for the reduction of hiPSC survival following DMSO treatment.

### Early Stages of hiPSC Differentiation Toward Intermediate Mesoderm

In this study, we investigated the effect of short-term DMSO pre-treatment on the early stages of kidney organoid differentiation from hiPSCs using the Morizane et al. protocol [21]. While DMSO is commonly associated with cytotoxic effects, we observed a reduction in cytotoxicity on day 2 of differentiation in cells pre-treated with DMSO, suggesting that short-term exposure may enhance cellular resilience during the initial differentiation phase. This effect was particularly evident in the 1% DMSO condition, which was also associated with improved mesoderm markers on day 4 of differentiation and sustained low level off-target lineage commitment.

As expected, the induction of primitive streak and intermediate mesoderm was initiated by treatment with CHIR99021 (a Wnt agonist) and Noggin (a BMP inhibitor), the former essential for mesoderm lineage specification and the latter represses lateral plate mesoderm differentiation [11]. Across all conditions, Wnt activation led to the downregulation of pluripotency markers such as NANOG, confirming appropriate exit from the pluripotent state. Notably, early germ layer markers revealed that SOX17, an endoderm marker, was upregulated in the negative control condition but not in either of the DMSO-treated groups, suggesting that DMSO may suppress off-target endodermal differentiation. Meanwhile, ectoderm markers PAX6 and SOX1 remained largely unchanged in all groups, indicating minimal ectodermal commitment during early differentiation. We also assessed potential off-target cardiac mesoderm induction by measuring NKX2.5 expression [52]. Although there was a transient increase in NKX2.5 from day 0 to day 2, expression declined from day 2 to day 4 in the 1% DMSO condition, indicating that any early cardiac lineage priming is not sustained and likely does not interfere with kidney specific differentiation outcomes.

Focusing on mesodermal specification, we found that primitive streak markers MIXL1 and TBXT were robustly upregulated across all conditions, confirming successful mesoderm induction [11, 53]. However, TBX6, a marker of paraxial mesoderm, remained largely unchanged, indicating that paraxial mesoderm was not significantly induced under these conditions. Additional mesoderm markers CDX1 and CDX2 showed transient upregulation, with CDX1 peaking on day 2 and CDX2 decreasing by day 4, patterns that mirror the dynamic nature of early mesodermal transitions in vivo. Interestingly, we observed a transient rise in FOXC1 expression from day 0 to day 2, followed by downregulation by day 4 in all conditions. Given its role in paraxial mesoderm and early mesodermal patterning, this suggests FOXC1 may participate in early fate priming but is not sustained through kidney-specific lineage progression. This is consistent with the observed lack of upregulation in TBX6, further supporting minimal commitment toward paraxial mesoderm [53]. To evaluate progression toward intermediate mesoderm fate, we assessed canonical markers including OSR1, LHX1, and WT1, which were detected at low levels across all conditions. However, PAX2 expression began to rise by day 4 specifically in the 1% DMSO group, suggesting a modest acceleration of intermediate mesoderm specification in this condition. However, we would expect higher co-expression with LHX1 in order to consider successful intermediate mesoderm induction [54]. While the expression remained low overall, this early upregulation could reflect improved responsiveness to Wnt and BMP modulation following DMSO pre-treatment.

Together, these findings suggest that 1% DMSO pre-treatment prior to initiating kidney organoid differentiation enhances the efficiency and specificity of early mesodermal and intermediate mesodermal induction, Likely by priming hiPSCs for Wnt signalling responsiveness and suppressing off-target Lineage commitment. Incorporating a low-dose 1% DMSO pre-treatment step may therefore serve as a simple and effective modification to improve the robustness and reproducibility of kidney organoid differentiation protocols.

### Metanephric Mesenchyme Nephron Progenitor Differentiation

In the second part of our study, we evaluated if the treatment of hiPSCs with DMSO had a downstream effect on the generation of MM progenitors, essential for kidney organoid differentiation. It is known that DMSO enhances differentiation of cells towards the three germ layers [17–19]. However, the influence of DMSO on the generation of MM progenitors and kidney organoids has not been reported. Hence, we evaluated the differentiation protocol efficiency by measuring the SIX2 + cells, a crucial MM NPC marker [55]. Previous studies demonstrated that it is critical to obtain 80–90% SIX2 + cells [11, 21] for successful downstream nephrogenesis. We quantified the difference in SIX2 + cells populations on day 9 of differentiation protocol and found that, on average, 1% DMSO had highest percentage of MM induction with 92% SIX2 + population, followed by highest cells retrieval rate, Likely due to higher proliferation of MM NPCs in the culture. Treating hiPSC with 1% DMSO was an effective way for creating a higher number of NPCs from a single batch differentiation culture. This would be particularly beneficial when generating many uniform 3D kidney organoids are needed for downstream applications (e.g., toxicity screening).

### Mature Kidney Organoid Nephron Differentiation

We also aimed to evaluate the success rate of kidney organoid generation in 96 well plate format, and structural composition of the resulting nephron development. Results showed that 1% DMSO treatment had the highest success rate of kidney organoid differentiation, followed by 2% DMSO, and lastly, non-treated hiPSCs.

Kidney organoids development for each condition showed similar structures with the expression of characteristic markers for different sections of the nephron: glomerulus (Podocalyxin and Nephrin), proximal tubule (LTL and Megalin), and distal tubule/collecting duct (E-cadherin and GATA3) [8, 10, 11, 22]. We observed that kidney organoids derived from hiPSCs treated with DMSO appear to have slightly higher protein expression of markers in the proximal tubule (megalin) and significantly higher expression of distal tubule/collecting duct (GATA3), whereas expression of markers for the glomerulus (podocalyxin) remained unchanged. These results suggest that treating hiPSCs with DMSO and subsequent differentiation following our optimised protocol resulting in kidney organoids with a higher number of tubular structures. GATA3 is not exclusively expressed in the distal tubule but also in the collecting duct arising from the ureteric bud lineage [10]. Therefore, it may be plausible that including DMSO treatment of hiPSCs leads to more cells differentiating into anterior intermediate mesoderm and subsequently differentiating towards the collecting duct. More analysis is needed to determine exactly which lineages emerge during the differentiation process [56]. These findings show that DMSO treatment enhances the differentiation of kidney organoids with developing nephron structures, particularly the distal tubule and collecting duct. By refining protocols with simple interventions, this work improves the efficiency of kidney organoid generation which can have significant impact on biomedical research and drug development.

## Conclusion and Future Perspectives

In this study, we demonstrated that short-term treatment of hiPSCs with low-dose DMSO enhances differentiation outcomes across three independent cell lines (LUMC, H101 and H107). Specifically, DMSO treatment led to the formation of a greater number of smaller, more circular colonies, morphological characteristics commonly associated with improved differentiation potential in hiPSC cultures. Importantly, this preconditioning strategy also improved the overall efficiency of kidney organoid generation using the Morizane et al. differentiation protocol [21], with 1% DMSO yielding the most effective enhancement in kidney organoid generation.

With a deeper analysis of our primary hiPSC Line LUMC we found that hiPSCs treated with 1–2% DMSO continue to express critical transcription genes and proteins essential for maintaining pluripotency. Furthermore, a low-dose DMSO treatment can modulate the cell cycle, stress response, and epigenetic landscape of hiPSCs without compromising their pluripotency. These alterations appear to poise cells in a more Lineage-competent state, potentially enhancing their responsiveness to directed differentiation. We also observed the emergence of colonies with shape, apico-basal polarity, and lumen formation in the centre of colonies indicative of epiblast spheroid morphology through immunofluorescent images of LUMC hiPSCs treated with 1–2% DMSO. Following kidney organoid differentiation, we showed the overall number of cells and percentage population expressing a critical metanephric mesenchyme marker, SIX2, in DMSO-treated LUMC hiPSCs compared to non-treated controls. 1% DMSO was found to give rise to the highest percentage of SIX2 + cells during differentiation and the highest success rate for fully mature organoids on day 21 of the differentiation protocol. Kidney organoids cultured until day 21 following the Morizane et al. differentiation protocol expressed characteristic markers for different segments of the developing nephron for each condition, with 2% DMSO-treated hiPSCs showing significantly higher expression of GATA3 + cells than non-treated control.

Incorporating short-term DMSO preconditioning strategies may help improve the efficiency and reproducibility of differentiation protocols, particularly in complex systems like kidney organoids. Our findings suggest that DMSO enhances differentiation potential, increasing the yield and scalability of tubular organoids, key factors for applications in drug screening and regenerative medicine, where large numbers of consistent organoids are often required. The morphological changes we report in hiPSCs following DMSO treatment also offer reproducible and quantifiable markers of preconditioning success, which is advantageous for standardization in high-throughput screening platforms. The improved differentiation protocol could be adapted to patient-specific hiPSC lines, enabling more consistent generation of kidney organoids for personalized medicine modeling. However, the potential epigenetic reprogramming and stress responses induced by DMSO also warrants caution, particularly in the context of translational clinical applications. Further investigation is needed to assess the long-term effects of DMSO on the epigenetic stability and functional integrity of hiPSCs and their derivatives.

Future work should explore multi-omics approaches to better characterize transcriptional, epigenetic, and proteomic changes, and assess the broader impact of DMSO on the culture environment, including potential interactions with medium components, growth factors, and basement membrane extract coatings. Such insights will be critical for refining preconditioning strategies and ensuring safety in translational applications. Additionally, incorporating DMSO pretreatment into other widely used kidney organoid protocols will help determine whether the observed effects are consistent and broadly applicable. In this study, we focused exclusively on the well-established Morizane et al. kidney organoid differentiation protocol to provide a clear and controlled assessment of DMSO preconditioning. Future work should incorporate DMSO pretreatment into other kidney organoid protocols to determine whether the observed enhancements in differentiation efficiency and reproducibility are broadly applicable across different systems. Such comparisons could yield further mechanistic insights and identify protocol-specific effects, supporting the development of optimized, versatile strategies for hiPSC-derived kidney modelling.

## Supplementary Information

Below is the link to the electronic supplementary material.

ESM 1 (MP4 1.47 MB)

ESM 2 (MP4 1.39 MB)

ESM 3 (MP4 1.49 MB)

ESM 4 (MP4 97.1 MB)

ESM 5 (MP4 76.4 MB)

ESM 6 (MP4 72.2 MB)

ESM 7 (DOCX 5.85 MB)

## Data Availability

All data supporting the findings of this study are available in DataverseNL at 10.34894/P4GM40.
