# Supplementary material for: Dimethyl Sulfoxide Conditions Induced Pluripotent Stem Cells for more Efficient Nephron Progenitor and Kidney Organoid Differentiation
Source: Stem Cell Rev Rep. 2025 Sep 18;21(8):2745–64. doi: 10.1007/s12015-025-10971-z (PMC12504399; doi:10.1007/s12015-025-10971-z)
Supplement: Supplementary file 7 — (DOCX 5.85 MB) [file 12015_2025_10971_MOESM7_ESM.docx]

**Dimethyl sulfoxide conditions induced pluripotent stem cells for more efficient nephron progenitor and kidney organoid differentiation**

Helen Kearney^1^, Aleksandra Rak-Raszewska^1^, Adrián Seijas-Gamardo^1^, Enrique Escarda-Castro^1^, Florian Caiment^2^, Paul Wieringa^1^, Lorenzo Moroni^1^, Carlos Mota^1^.

^1^ *MERLN Institute for Technology-Inspired Regenerative Medicine, Maastricht University, 6229 ER, Maastricht, the Netherlands*

^2^ *Translational Genomics, GROW Research Institute for Oncology and Reproduction, Maastricht University, 6229 ER, Maastricht, the Netherlands*

# **Supplementary Information**

Table S1: List of antibodies for flow cytometry, immunofluorescence images and in-cell western.

| **Antibody name** | **Host** | **Dilution** | **Manufacturer** | **Catalog #** |
| --- | --- | --- | --- | --- |
| TRA-1-81 | Mouse | 1:10 | BD bioscience | 560161 |
| TRA-1-60 | Mouse | 1:10 | BD bioscience | 560193 |
| SSEA3 | Rat | 1:40 | BD bioscience | 561145 |
| SSEA4 | Mouse | 1:40 | BD bioscience | 560219 |
| SOX2-PE | human | 1:100 | Miltenyi Biotech | 130-121-053 |
| OCT3/4-AF647 | Mouse | 1:100 | BD Bioscience | 560329 |
| SOX2 | Mouse | 1:200 | Abcam | ab79351 |
| OCT4 | Rabbit | 1:250 | Abcam | ab200834 |
| Podocalyxin | Goat | 1:200 | R&D systems | AF1658 |
| Beta-catenin | Rabbit | 1:200 | Abcam | ab2365 |
| ZO-1 | Mouse | 1:200 | Invitrogen | 33-9100 |
| Phallodin | n/a | 1:200 | Invitrogen | 10125092 |
| Six homeobox 2 | Rabbit | 1:300 | Proteintech | 11562-1-AP |
| PAX2 | Goat | 1:100 | R&D systems | AF3364 |
| Lotus tetragonolobus lectin | n/a | 1:300 | Vector laboratories | FL-1321-2 |
| E-cadherin | Mouse | 1:150 | BD bioscience | 610181 |
| Megalin | Mouse | 1:300 | R&D Systems | MAB9578-100 |
| Nephrin | Sheep | 1:300 | R&D systems | AF4269 |
| GATA3 | Rabbit | 1:500 | Cell signalling | 5852S |
| 4′,6-diamidino-2-phenylindole | n/a | 2ng/mL | Sigma-Aldrich | 32670 |
| Donkey anti-goat AF568 | Donkey | 1:1000 | Invitrogen | 10463972 |
| Donkey anti-mouse AF647 | Donkey | 1:1000 | Invitrogen | 15980296 |
| Donkey anti-sheep AF568 | Donkey | 1:1000 | Invitrogen | A21099 |
| Donkey anti-rabbit AF488 | Donkey | 1:1000 | Invitrogen | 10424752 |
| Donkey anti-goat AF647 | Donkey | 1:1000 | Invitrogen | 10493402 |
| Goat anti-mouse AF647 | Goat | 1:500 | Invitrogen | A-21240 |
| Goat anti-mouse AF568 | Goat | 1:1000 | Invitrogen | A-21245 |
| Goat anti-mouse IRDye 800CW | Goat | 1:500 | LICOR | 926-32210 |
| Goat anti-rabbit IRDye 800CW | Goat | 1:500 | LICOR | 926-32211 |
| Donkey anti-goat IRDye 800CW | Donkey | 1:500 | LICOR | 926-32214 |

Table S2: List of primers used for qPCR.

| **Gene ID** | **Forward** | **Reverse** |
| --- | --- | --- |
| ATP5PB | TTTCATACAGGGCAGCCACA | AAGCCCAGTTCCGAGTACAT |
| SOX2 | GCTTAGCCTCGTCGATGAAC | AACCCCAAGATGCACAACTC |
| OCT4 | GGTTCTCGATACTGGTTCGC | GTGGAGGAAGCTGACAACAA |
| c-myc | CCTGGTGCTCCATGAGGAGAC | CAGACTCTGACCTTTTGCCAGG |
| Nanog | ACCAGTCCCAAAGGCAAACA | TCTGCTGGAGGCTGAGGTAT |
| HSPA5 | CTGTCCAGGCTGGTGTGCTCT | CTTGGTAGGCACCACTGTGTTC |
| HSP90B1 | GGAGAGTCGTGAAGCAGTTGAG | CCACCAAAGCACACGGAGATTC |
| Vimentin | GCCGAAAACACCCTGCAATC | TCCTGGATTTCCTCTTCGTGG |
| CDH2 | CCTCCAGAGTTTACTGCCATGAC | GTAGGATCTCCGCCACTGATTC |
| PTK2 | GCCTTATGACGAAATGCTGGGC | CCTGTCTTCTGGACTCCATCCT |
| CD151 | GGAGAACCTGAAGGACACCATG | CAGTCCTGTGAGTTGTTGCTGC |
| ITGa5 | GCCGATTCACATCGCTCTCAAC | GTCTTCTCCACAGTCCAGCAAG |
| ITGa6 | CGAAACCAAGGTTCTGAGCCCA | CTTGGATCTCCACTGAGGCAGT |
| ITGaV | AGGAGAAGGTGCCTACGAAGCT | GCACAGGAAAGTCTTGCTAAGGC |
| ITGb1 | GGATTCTCCAGAAGGTGGTTTCG | TGCCACCAAGTTTCCCATCTCC |
| ITGb5 | GCCTTTCTGTGAGTGCGACAAC | CCGATGTAACCTGCATGGCACT |
| PODXL | AACCCGGCCCAAGATAAGTG | TTGGCAGGGAGCTTAGTGTG |
| β-Catenin | CACAAGCAGAGTGCTGAAGGTG | GATTCCTGAGAGTCCAAAGACAG |
| SOX17 | ACGCTTTCATGGTGTGGGCTAAG | GTCAGCGCCTTCCACGACTTG |
| PAX6 | CTGAGGAATCAGAGAAGACAGGC | ATGGAGCCAGATGTGAAGGAGG |
| SOX1 | GAGTGGAAGGTCATGTCCGAGG | CCTTCTTGAGCAGCGTCTTGGT |
| TBXT | AGGTACCCAACCCTGAGGA | GCAGGTGAGTTGTCAGAATAGGT |
| MIXL1 | ACGTCTTTCAGCGCCGAACAG | TTGGTTCGGGCAGGCAGTTCA |
| TBX6 | CAGAAGCTGTCGGACTCACC | CCCAGGGACGGGTACAATTC |
| NKX2.5 | AAGTGTGCGTCTGCCTTTCCCG | TTGTCCGCCTCTGTCTTCTCCA |
| CDX1 | GAGAAGGAGTTTCATTACAGCCG | GTTCACTTTGCGCTCCTTTGCC |
| CDX2 | ACAGTCGCTACATCACCATCCG | CCTCTCCTTTGCTCTGCGGTTC |
| FOXC1 | ACATGTTGTAGGAGTCCGGG | CTCAACGAGTGCTTCGTCAA |
| OSR1 | GACATCTGCCACAAAGCCTTC | CCCACAGGTTCTATTTAGCATTTGA |
| LHX1 | ACCAGGTCGCTAGGGGAG | TCCAGGGAAGGCAAACTCTA |
| HOXD11 | GCCAGTGTGCTGTCGTTCCC | CTTCCTACAGACCCCGCCGT |
| PAX2 | CAAAGTTCAGCAGCCTTTCC | CCACACCACTCTGGGAATCT |
| Wt1 | TTTCCTAACGCGCCCTACC | TGACCGTGCTGTAACCCTG |


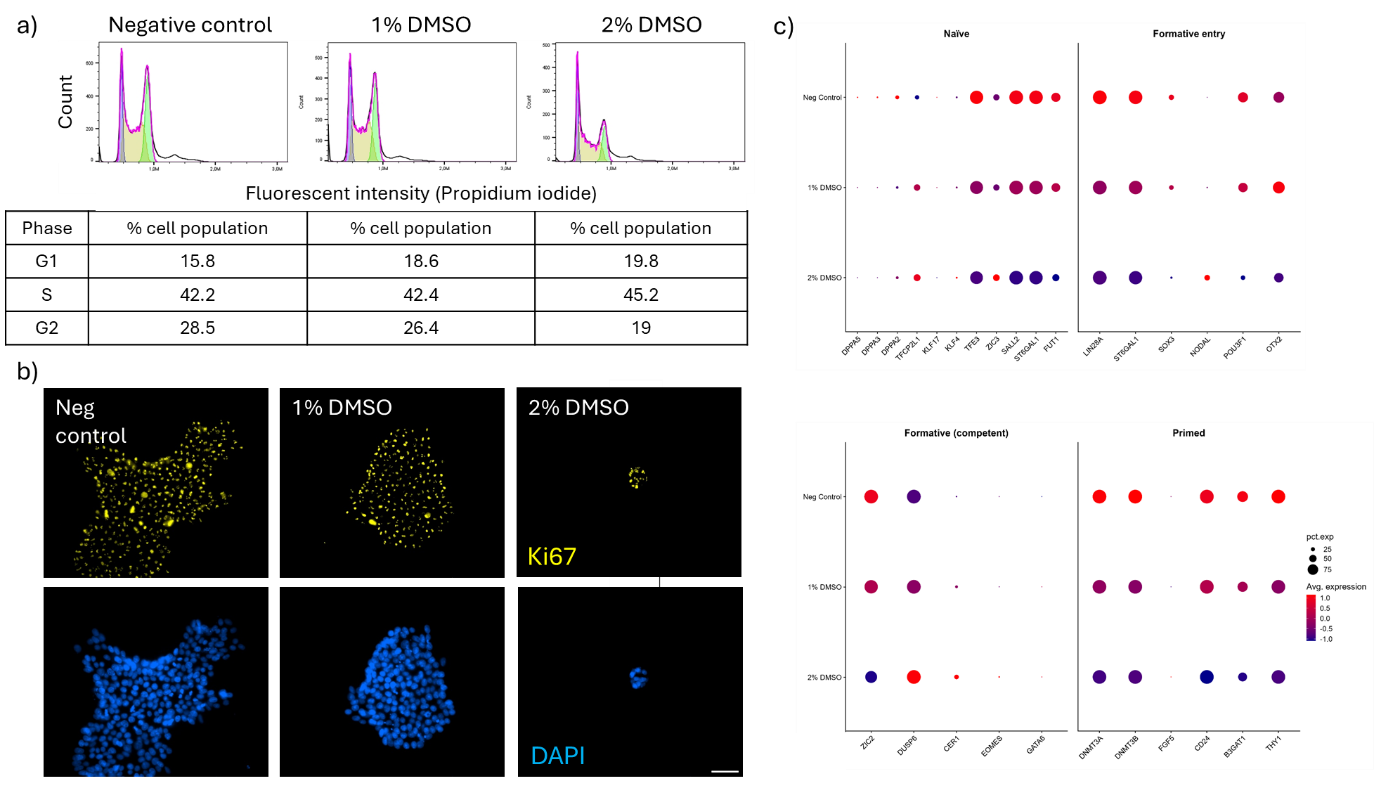


**Figure S1 –** a) FlowJo software graphs presenting cell cycle analysis used to quantify % LUMC hiPSCs in G0/G1 phase (N=3). b) Immunofluorescent images of LUMC hiPSC colonies expressing Ki67 and stained with DAPI (N=2), scale bar – 50 µm. c) RNA-seq dot plots showing the expression levels and percentage of LUMC hiPSCs expressing genes associated with naïve, formative and primed state pluripotency following DMSO treatment for 24hr (N=1).

**
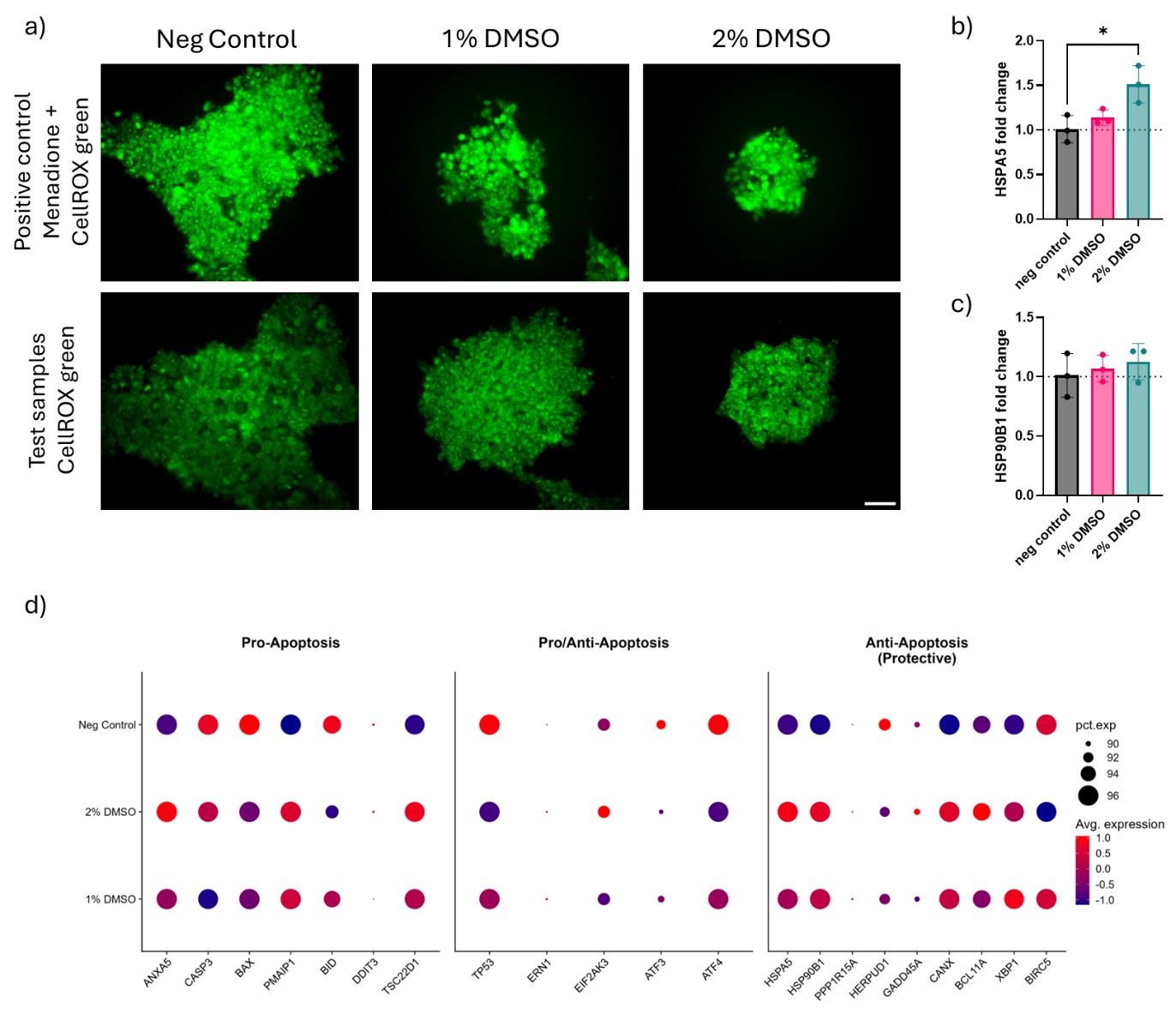
**

**Figure S2 –** a) CellROX green assay showing reactive oxygen species (ROS) activity in LUMC hiPSCs treated with DMSO for 24hr (n=1), scale bar - 50 µm. LUMC hiPSC gene expression analysis by qPCR of heat shock proteins; b) HSPA5 and c) HSP90B1 following DMSO treatment for 24hr (n=3). d) RNA-seq dot plots showing the expression levels and percentage of LUMC hiPSCs expressing genes associated with necrosis and apoptosis following DMSO treatment for 24hr (N=1).


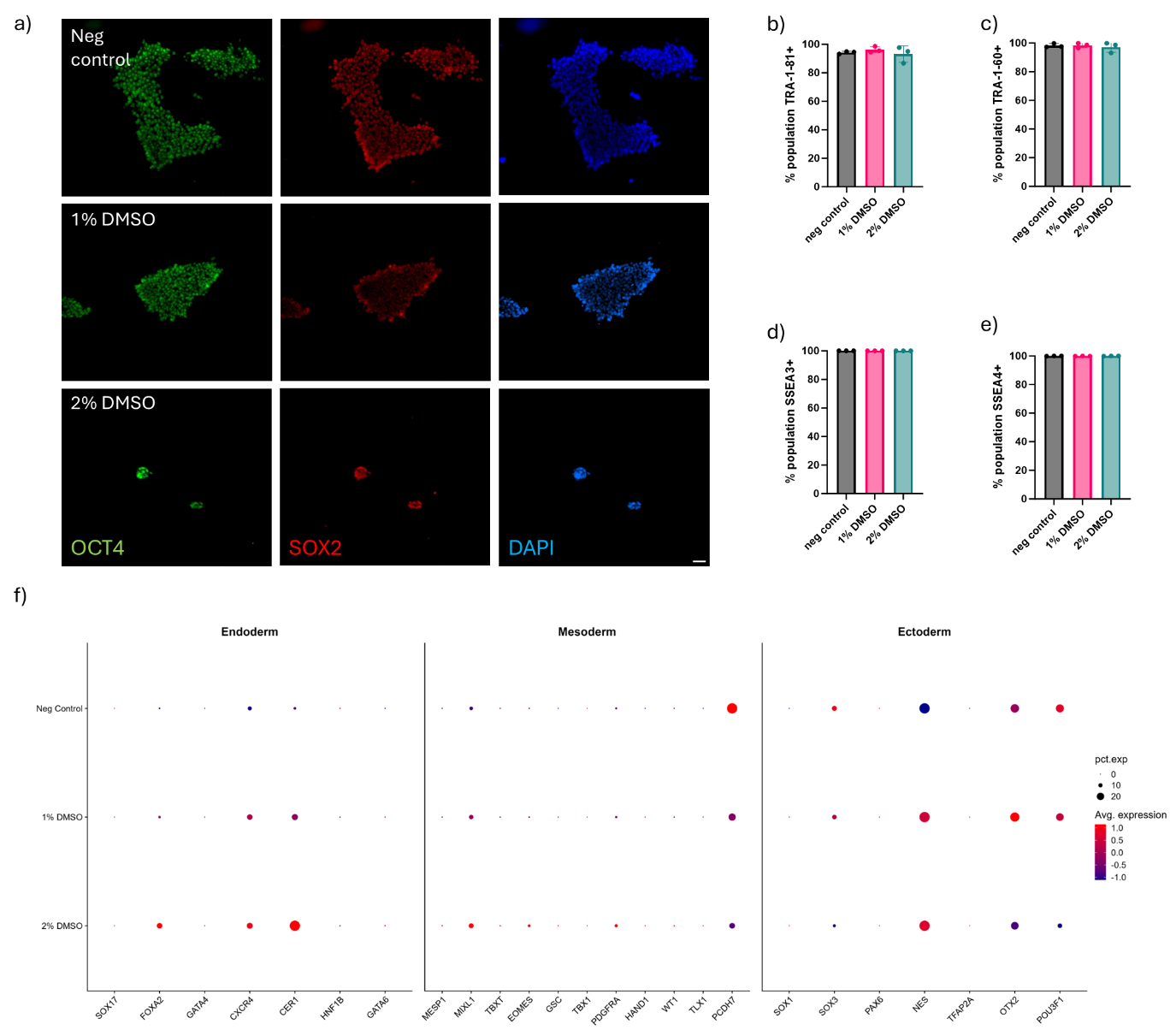


**Figure S3 –** a) Immunofluorescent images of LUMC hiPSC colonies expressing OCT4 and SOX2 and stained with DAPI following DMSO treatment for 24hr, scale bar – 50 µm. The percentage LUMC hiPSCs expressing pluripotent cell surface markers; b) TRA-1-81, c) TRA-1-60, d) SSEA3 and e) SSEA4 measured by flow cytometry following DMSO treatment for 24hr (N=3). f) RNA-seq dot plots showing the expression levels and percentage of LUMC hiPSCs expressing genes associated with three different germ layers: endoderm, mesoderm and ectoderm, following DMSO treatement for 24hr (n=1).

Immunofluorescent images of hiPSC colonies showed OCT4, and SOX2 protein expression throughout the hiPSC colonies (Figure S3a). Flow cytometry results for SSEA3, SSEA4, TRA-1-60, and TRA-1-81 (Figure S3b-e) showed an overall high expression of characteristic pluripotent cell surface markers for all conditions. A significant decrease in protein expression of critical pluripotent transcription factors OCT4 and SOX2 (Figure Sf-g) was observed following 2% DMSO treatment.


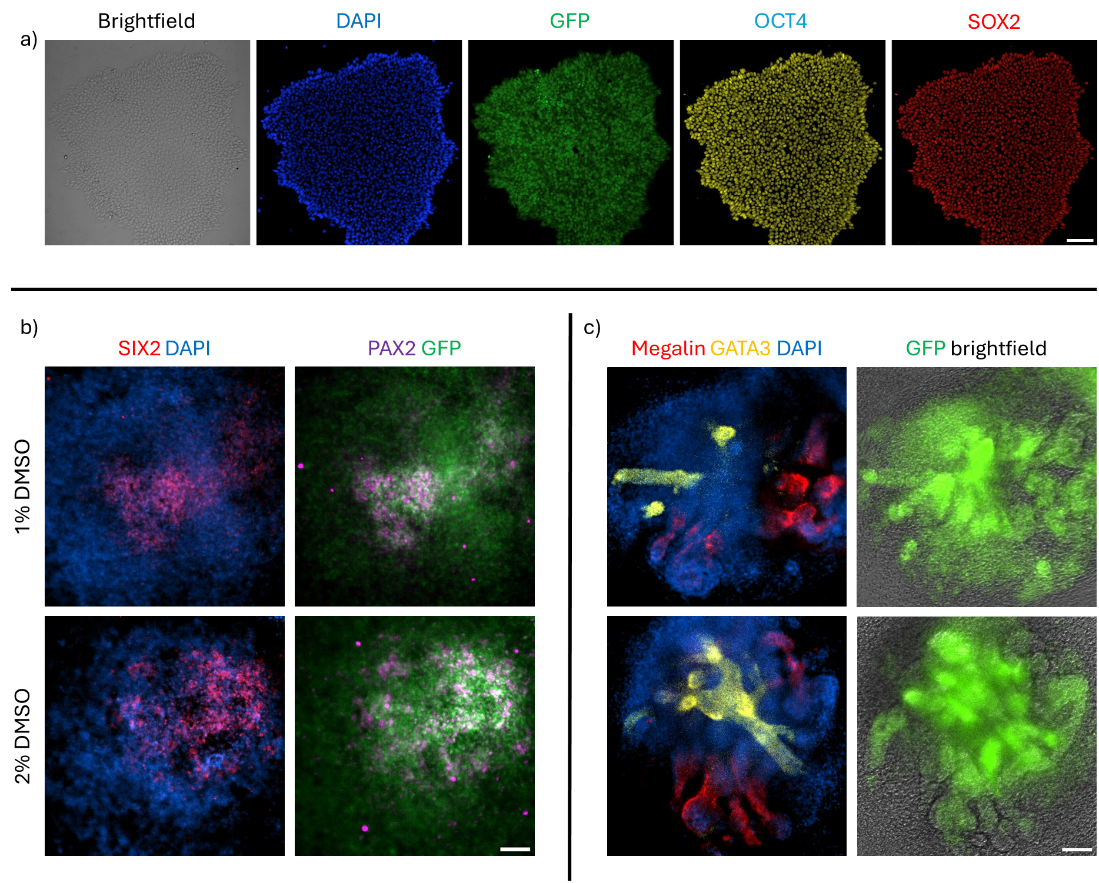


**Figure S4 –** Immunofluorescent images of a) LUMC-GFP+ hiPSCs colony (green) stained for markers of pluripotency OCT4 (yellow) and SOX2 (red), and nuclear DAPI (blue) staining. LUMC-GFP+ hiPSCs differentiated towards b) MM nephron progenitors expressing SIX2 (red), PAX2 (magenta), and nuclear DAPI (blue) staining on day 9 of kidney organoid differentiation, and c) LUMC-GFP+-derived kidney organoids expressing nephron markers Megalin (red), GATA3 (yellow), and nuclear DAPI (blue) staining on day 21 of kidney organoid differentiation (N=1). Scale bar – 100 µm. GFP intensity was found to be higher in the densely packed organoids and therefore microscope exposure settings were set to favour visualization of these areas.


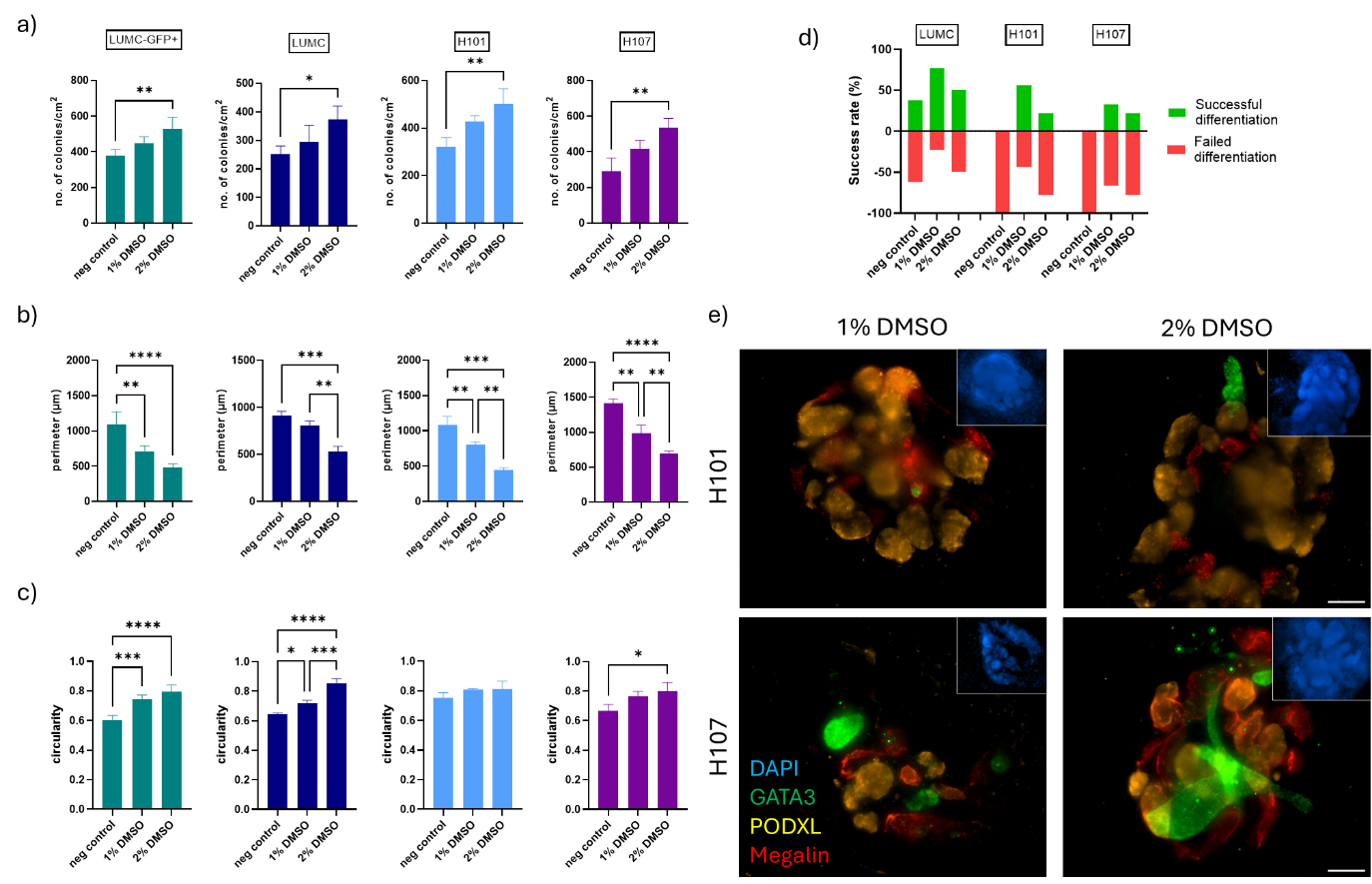


**Figure S5 –** Parametric analysis of four different hiPSC lines (LUMC-GFP+, LUMC, H101 and H107) treated with 0-2% DMSO for 24hr prior to kidney organoid differentiation. 24hr timepoint of LUMC-GFP+ live cell analysis, and nuclei staining of cells in colonies fixed in 4% PFA were imaged and used to a) count the number of cell colonies, b) measure the perimeter of cell colonies, and c) cell colony circularity; LUMC-GFP+ (N=3), LUMC (n=3), H101 (n=3) and H107 (n=3). On day 21 of kidney organoid differentiation the d) success rate of kidney organoid differentiation for the three different hiPSC lines was assessed in each well of 96 well plate, LUMC (N=9), H101(N=3) and H107 (N=3). e) Immunofluorescent images confirming expression of nephron markers in kidney organoid structures from differentiation cultures using H101 (n=1) and H107 (n=1); Podocalyxin (yellow), Megalin (red) and GATA3 (green) (n=1), scale bar – 100 µm.


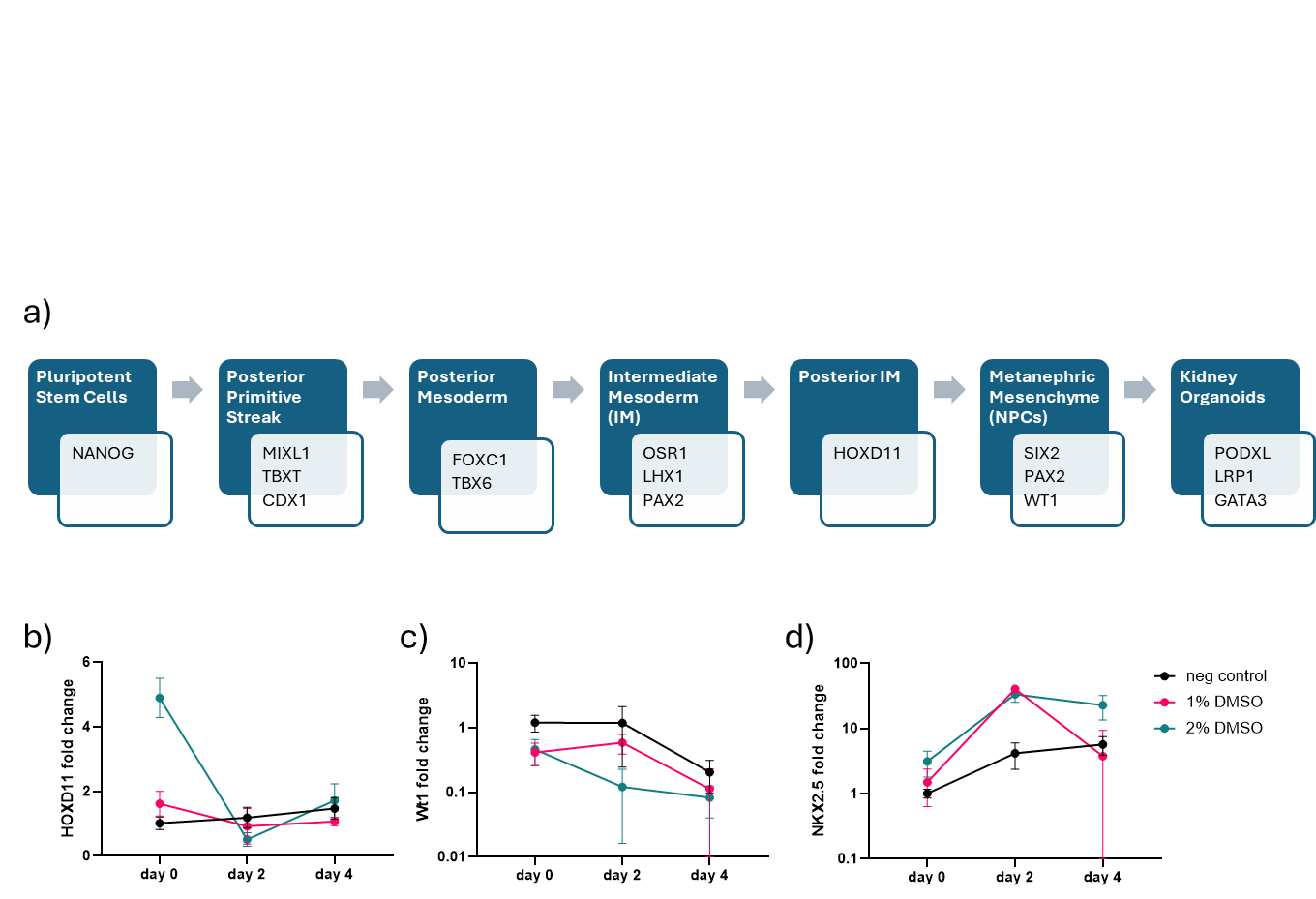


**Figure S6 –** a) Schematic of gene expression map throughout different stages of kidney organoid development. LUMC hiPSC gene expression analysis by qPCR during mesoderm induction over first four days of kidney organoid differentiation protocol; b) Wt1, c) HOXD11, and d) NKX2.5 (n=3).


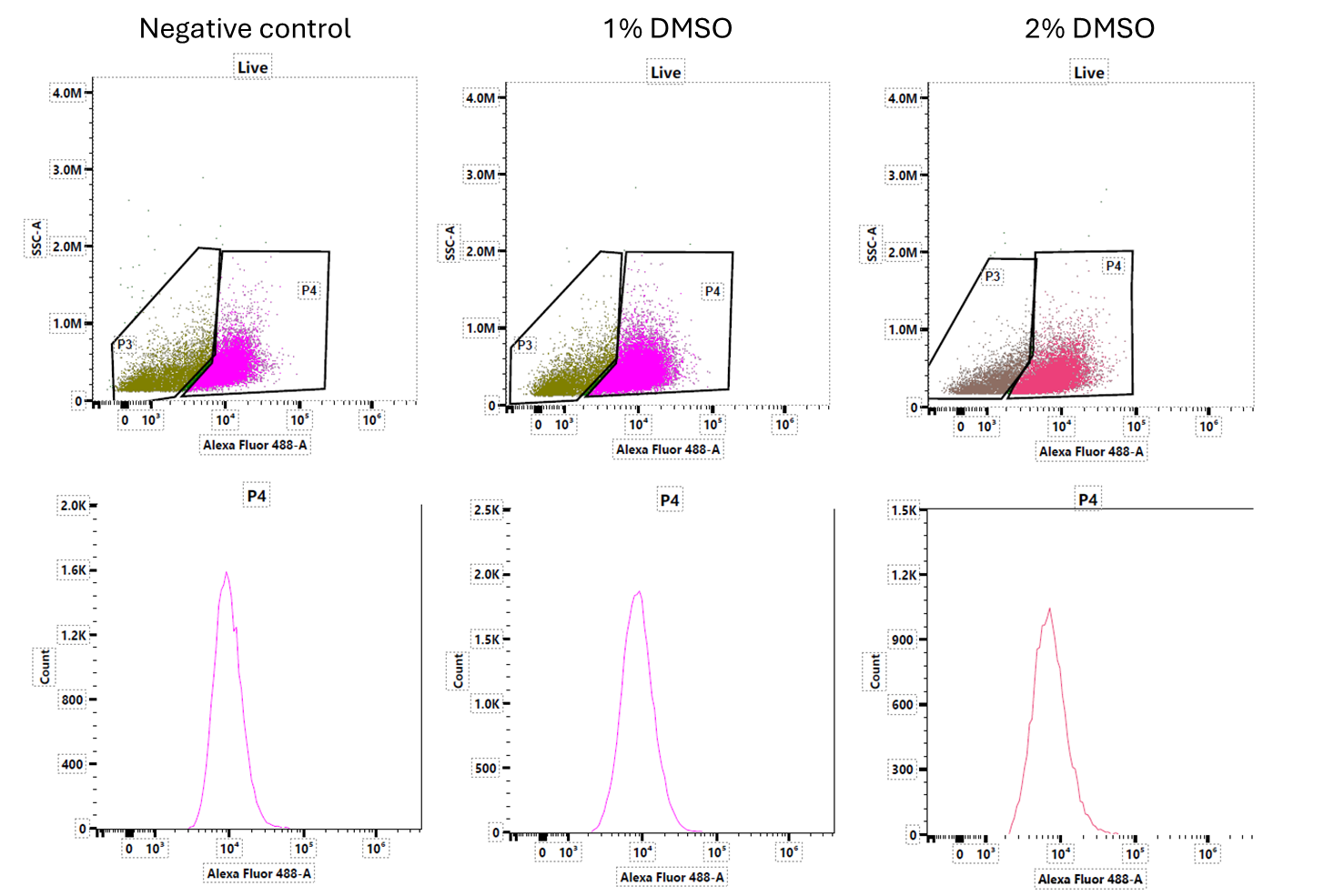


**Figure S7 –** Flow cytometry analysis of single-cell cloud plots comparing side scatter versus fluorescence intensity and count versus fluorescence intensity for SIX2+ cells in LUMC hiPSC-derived nephron progenitor population on day 9 of kidney organoid differentiation protocol (N=5).


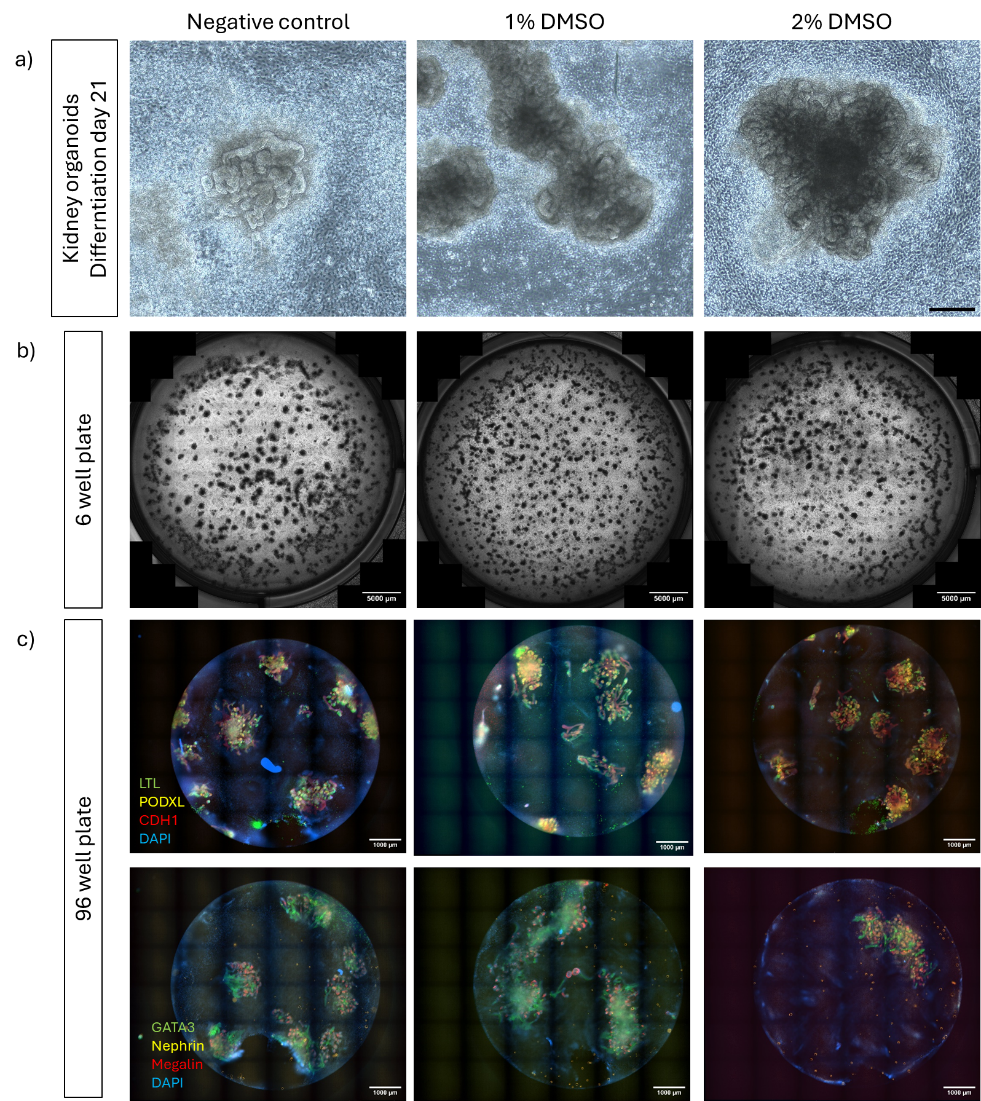


**Figure S8 –** Brightfield images of a) LUMC hiPSC-derived kidney organoids differentiated until day 21, scale bar - 100 µm, b) stitched image of an entire well in a 6-well plate containing LUMC hiPSC-derived kidney organoids differentiated until day 21, scale bar - 5000 µm. c) Stitched immunofluorescence image of an entire well in a 96-well plate with LUMC hiPSC-derived kidney organoids differentiated until day 21, scale bar - 1000 µm.


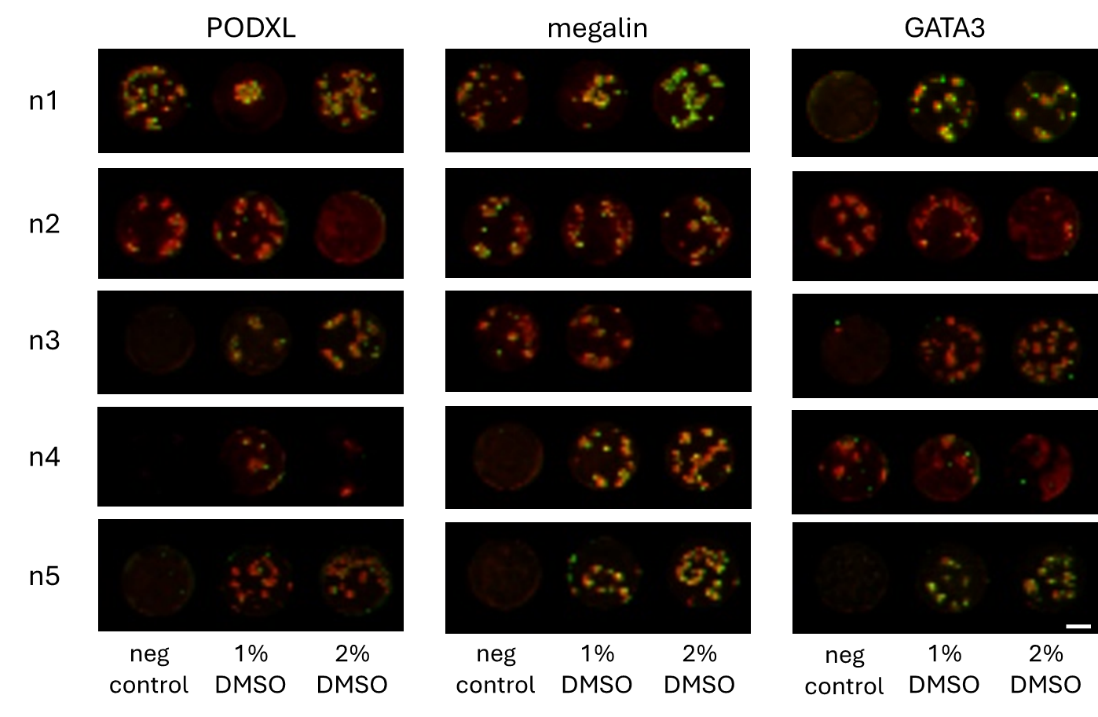


**Figure S9 –** LICOR NIR images of wells used for quantifying total protein in LUMC hiPSC-derived kidney organoids - CellTag700 (red) and Podocalyxin, Megalin and GATA3 (green). Scale bar – 200 µm

**Video S1a –** Timelapse video of LUMC-GFP+ colonies under brightfield of non-treated control well (negative control). Single images taken every 20 min over 24 hr.

**Video S1b –** Timelapse video of LUMC-GFP+ colonies under brightfield treated with 1% DMSO. Single images taken every 20 min over 24 hr.

**Video S1c –** Timelapse video of LUMC-GFP+ colonies under brightfield treated with 2% DMSO. Single images taken every 20 min over 24 hr.

**Video S2a –** Timelapse video of LUMC colonies under brightfield non-treated control well (negative control). Single images taken every 20 min over 24 hr.

**Video S2b –** Timelapse video of LUMC colonies under brightfield treated with 1% DMSO. Single images taken every 20 min over 24 hr.

**Video S2c –** Timelapse video of LUMC colonies under brightfield treated with 2% DMSO. Single images taken every 20 min over 24 hr.
